# Supplementary material for: Selection for Reducing Energy Cost of Protein Production Drives the GC Content and Amino Acid Composition Bias in Gene Transfer Agents
Source: mBio. 2020 Jul 14;11(4):e01206-20. doi: 10.1128/mBio.01206-20 (PMC7360931; doi:10.1128/mBio.01206-20)
Supplement: TABLE S1 [file mBio.01206-20-st001.pdf]

Table S1. The number of GTA genes detected in 212 alphaproteobacterial genomes and viruses from the RefSeq database.

GTA gene	Number of GTA genes	Number of viral homologs	Used for cost of production analysis	Used for ancestral reconstructions	Functional annotation[#]
g1	41	0			hypothetical protein
g2	205	16	X	X	ATP-binding protein
g3	204	261	X	X	phage-portal protein
g3.5	57	0			hypothetical protein
g4	205	164	X	X	HK97 family phage prohead protease
g5	205	137	X	X	phage major capsid protein
g6	209	36	X	X	hypothetical protein
g7	151	63	X	X	head-tail adaptor protein
g8	209	66	X	X	DUF3168 domain-containing protein
g9	209	18	X	X	phage major tail protein, TP901-1 family
g10	208	1		X	gene transfer agent family protein
g10.1	203	0			phage tail assembly chaperone
g11	209	1		X	phage tail tape measure protein
g12	209	26	X	X	TIGR02217 family protein
g13	207	70	X	X	DUF2163 domain-containing protein
g14	200	26	X	X	peptidase
g15	200	12	X	X	hypothetical protein

[#] Taken from the RefSeq record NC_014034.1
