# Supplementary figures and images for: Selection for Reducing Energy Cost of Protein Production Drives the GC Content and Amino Acid Composition Bias in Gene Transfer Agents
Source: mBio. 2020 Jul 14;11(4):e01206-20. doi: 10.1128/mBio.01206-20 (PMC7360931; doi:10.1128/mBio.01206-20)

Relative abundance of amino acids encoded by GC-rich codons

0.45

0.40

0.35

0.30

Origin

Genome

GTA region

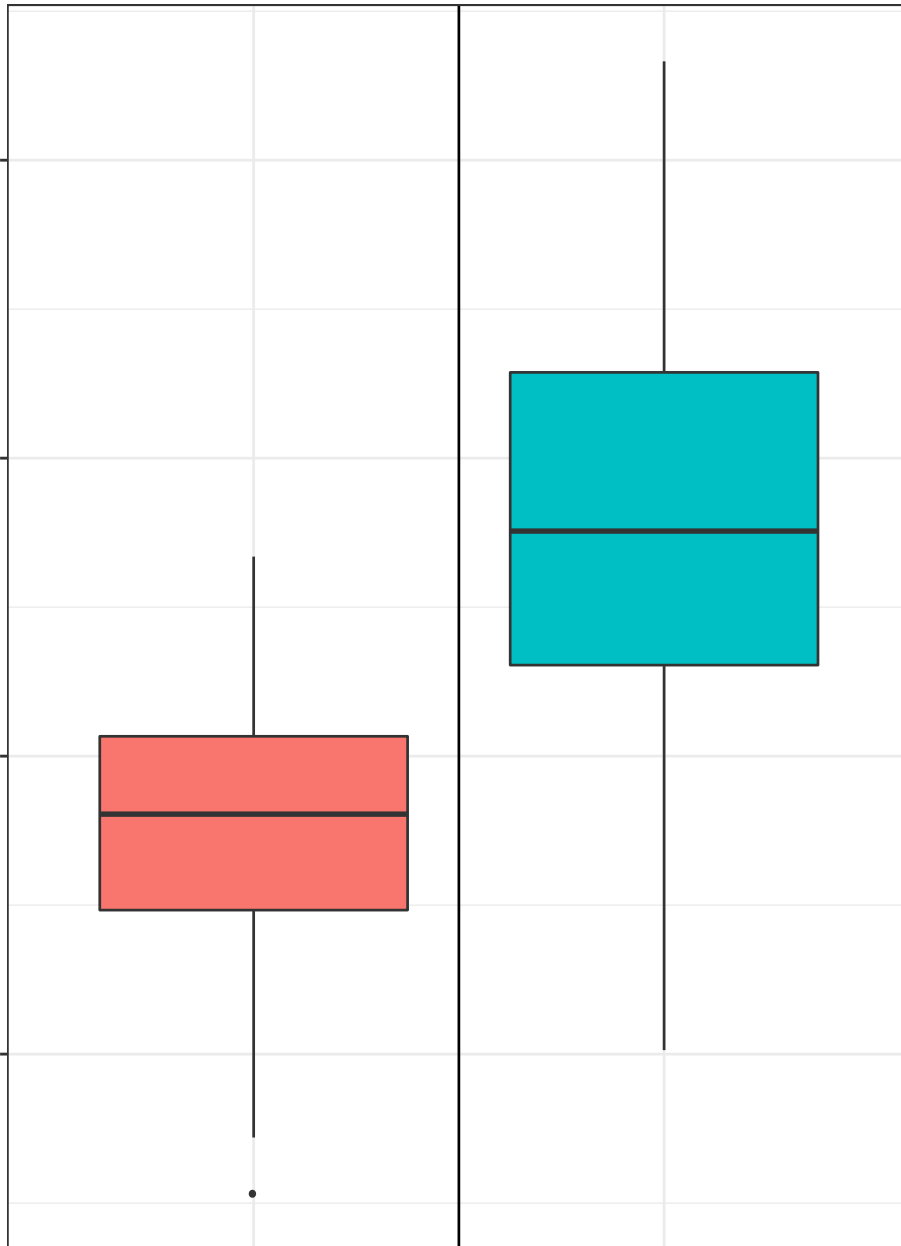

Supplement: FIG S1 [file mBio.01206-20-sf001.pdf]

Amino acid biosynthetic pathway

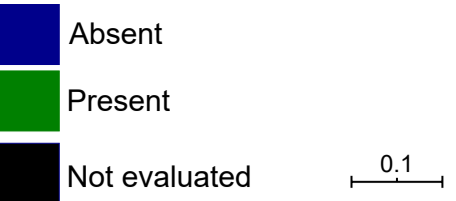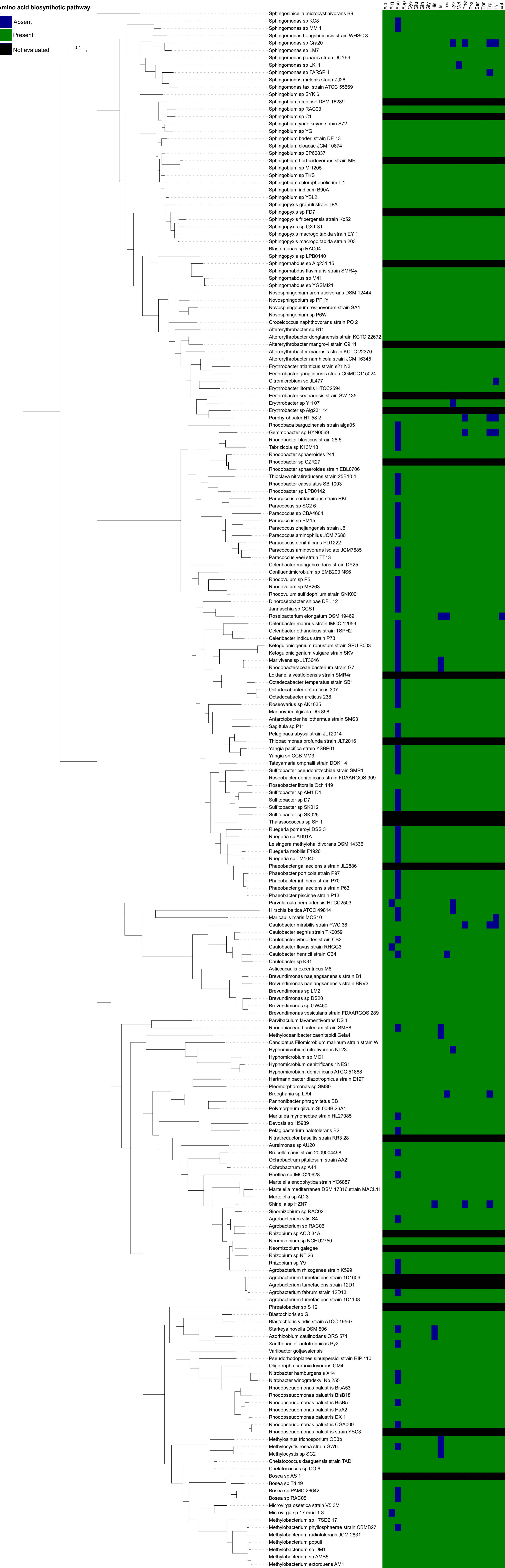

Supplement: FIG S2 [file mBio.01206-20-sf002.pdf]

g2

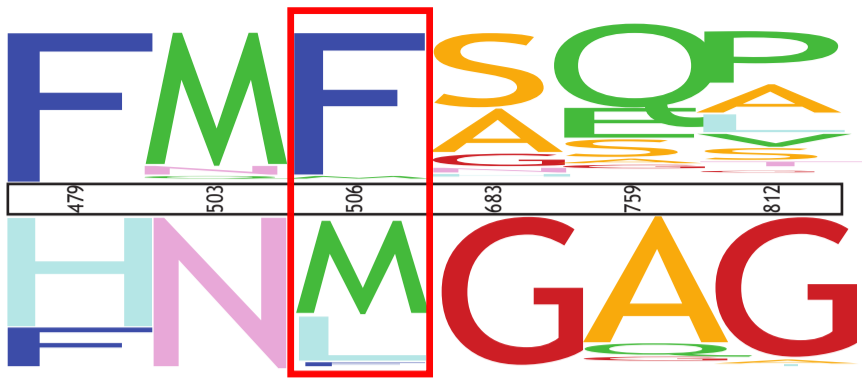

g3

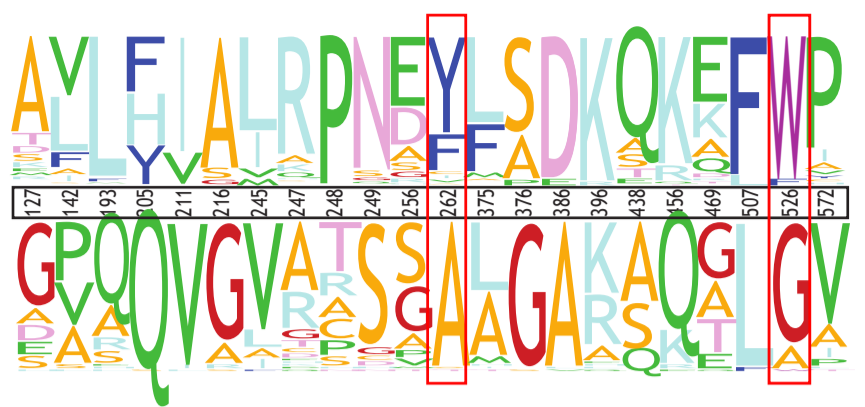

g4

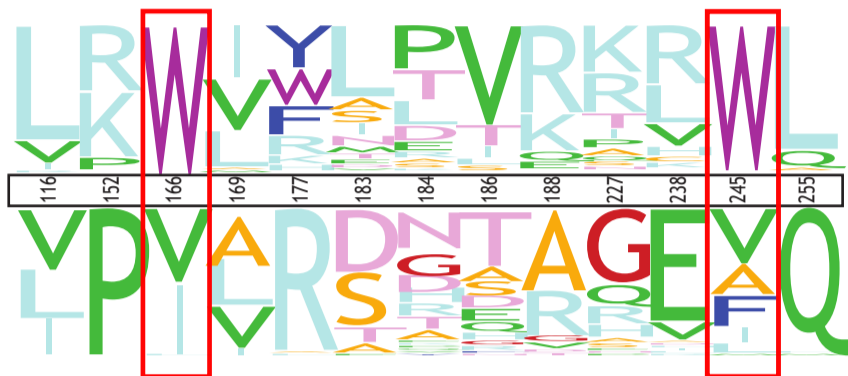

g5

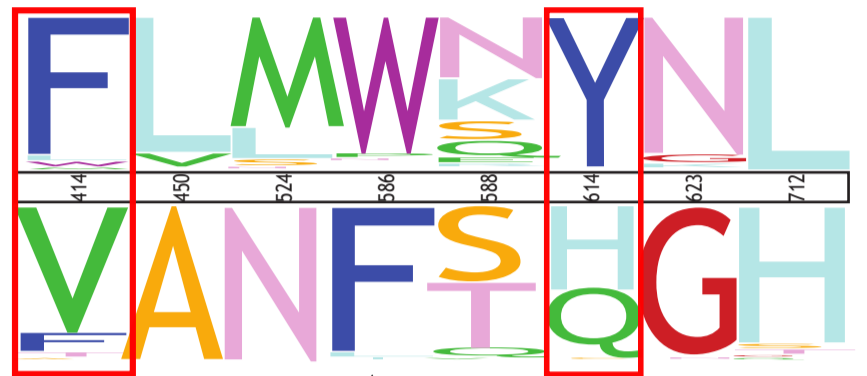

g6

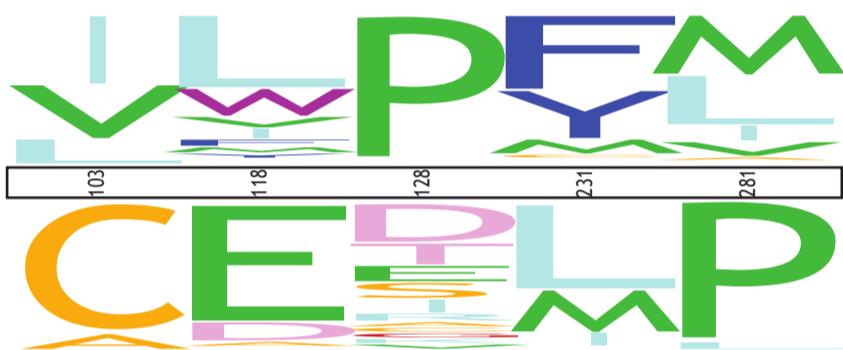

g9

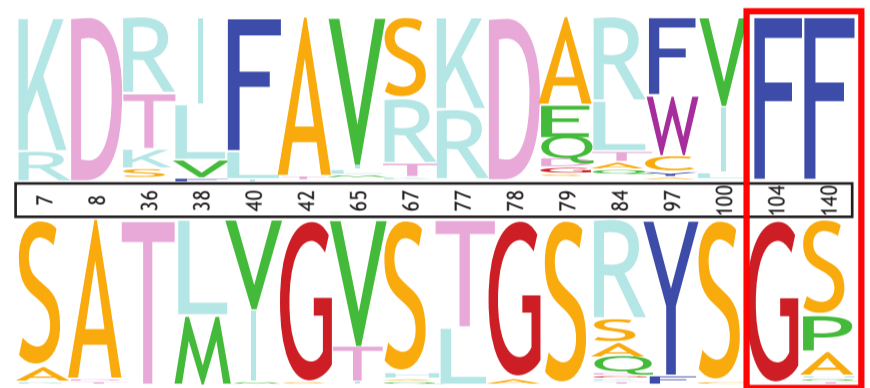

g12

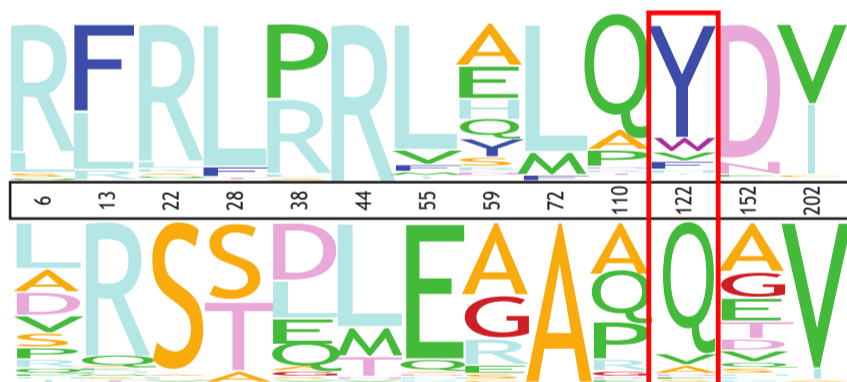

g13

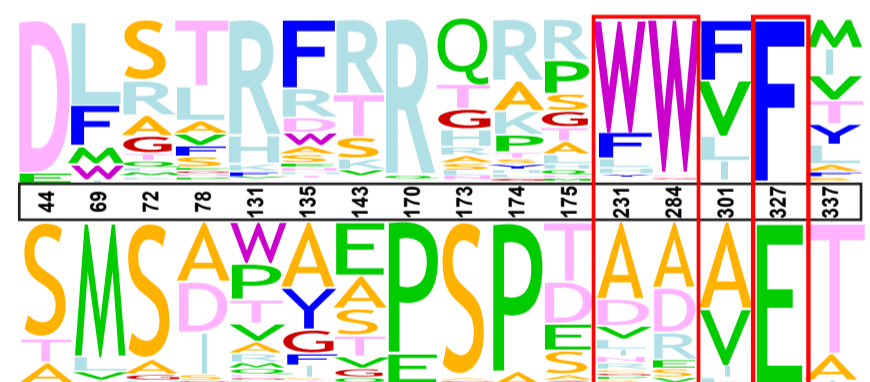

g15

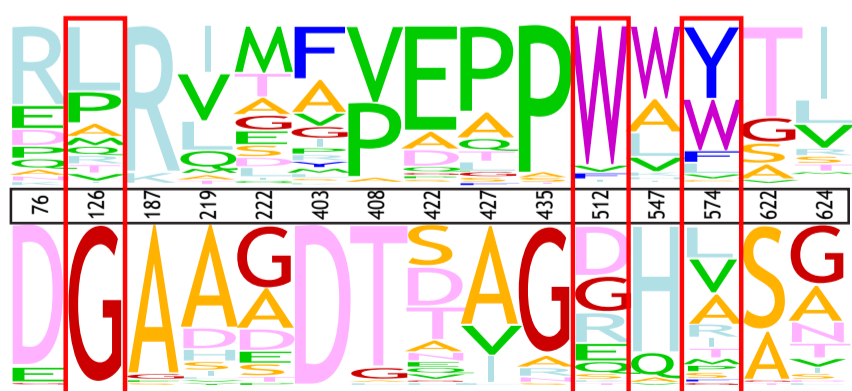

Number of carbons per side chain per amino acid

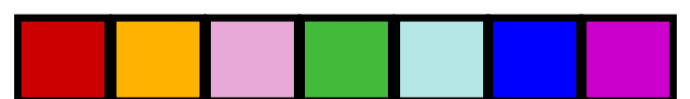

0 1 2 3 4 7 9

Supplement: FIG S5 [file mBio.01206-20-sf005.pdf]
