# Supplementary material for: Selection for Reducing Energy Cost of Protein Production Drives the GC Content and Amino Acid Composition Bias in Gene Transfer Agents
Source: mBio. 2020 Jul 14;11(4):e01206-20. doi: 10.1128/mBio.01206-20 (PMC7360931; doi:10.1128/mBio.01206-20)
Supplement: FIG S3 [file mBio.01206-20-sf003.pdf]

g6

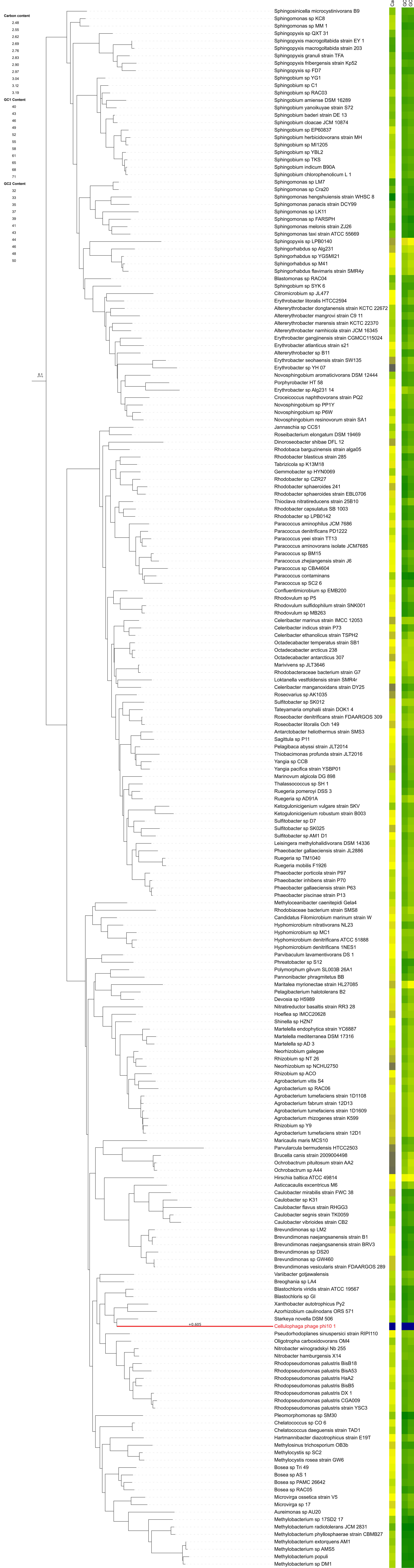
Carbon content
2.48
2.55
2.62
2.69
2.76
2.83
2.90
2.97
3.04
3.12
3.19
GC1 Content
40
43
46
49
52
55
58
61
65
68
71
GC2 Content
32
33
35
37
39
41
43
44
46
48
50
0.1
+0.605
Carbon content
GC1 Content
GC2 Content
Sphingosinicella microcystinivorans B9
Sphingomonas sp KC8
Sphingomonas sp MM 1
Sphingopyxis sp QXT 31
Sphingopyxis macrogoltabida strain EY 1
Sphingopyxis macrogoltabida strain 203
Sphingopyxis granuli strain TFA
Sphingopyxis fribergensis strain Kp52
Sphingopyxis sp FD7
Sphingobium sp YG1
Sphingobium sp C1
Sphingobium sp RAC03
Sphingobium amiense DSM 16289
Sphingobium yanoikuyae strain S72
Sphingobium baderi strain DE 13
Sphingobium cloacae JCM 10874
Sphingobium sp EP60837
Sphingobium herbicidovorans strain MH
Sphingobium sp MI1205
Sphingobium sp YBL2
Sphingobium sp TKS
Sphingobium indicum B90A
Sphingobium chlorophenolicum L 1
Sphingomonas sp LM7
Sphingomonas sp Cra20
Sphingomonas hengshuiensis strain WHSC 8
Sphingomonas panacis strain DCY99
Sphingomonas sp LK11
Sphingomonas sp FARSPH
Sphingomonas melonis strain ZJ26
Sphingomonas taxi strain ATCC 55669
Sphingopyxis sp LPB0140
Sphingorhabdus sp Alg231
Sphingorhabdus sp YGSMI21
Sphingorhabdus sp M41
Sphingorhabdus flavimaris strain SMR4y
Blastomonas sp RAC04
Sphingobium sp SYK 6
Citromicrobium sp JL477
Erythrobacter litoralis HTCC2594
Altererythrobacter dongtanensis strain KCTC 22672
Altererythrobacter mangrovi strain C9 11
Altererythrobacter marensis strain KCTC 22370
Altererythrobacter namhicola strain JCM 16345
Erythrobacter gangjinensis strain CGMCC115024
Erythrobacter atlanticus strain s21
Altererythrobacter sp B11
Erythrobacter seohaensis strain SW135
Erythrobacter sp YH 07
Novosphingobium aromaticivorans DSM 12444
Porphyrobacter HT 58
Erythrobacter sp Alg231 14
Croceicoccus naphthovorans strain PQ2
Novosphingobium sp PP1Y
Novosphingobium sp P6W
Novosphingobium resinovorum strain SA1
Jannaschia sp CCS1
Roseibacterium elongatum DSM 19469
Dinoroseobacter shibae DFL 12
Rhodobaca barguzinensis strain alga05
Rhodobacter blasticus strain 285
Tabrizicola sp K13M18
Gemmobacter sp HYN0069
Rhodobacter sp CZR27
Rhodobacter sphaeroides 241
Rhodobacter sphaeroides strain EBL0706
Thioclava nitratireducens strain 25B10
Rhodobacter capsulatus SB 1003
Rhodobacter sp LPB0142
Paracoccus aminophilus JCM 7686
Paracoccus denitrificans PD1222
Paracoccus yeei strain TT13
Paracoccus aminovorans isolate JCM7685
Paracoccus sp BM15
Paracoccus zhejiangensis strain J6
Paracoccus sp CBA4604
Paracoccus contaminans
Paracoccus sp SC2 6
Confluentimicrobium sp EMB200
Rhodovulum sp P5
Rhodovulum sulfidophilum strain SNK001
Rhodovulum sp MB263
Celeribacter marinus strain IMCC 12053
Celeribacter indicus strain P73
Celeribacter ethanolicus strain TSPH2
Octadecabacter temperatus strain SB1
Octadecabacter arcticus 238
Octadecabacter antarcticus 307
Marivivens sp JLT3646
Rhodobacteraceae bacterium strain G7
Loktanella vestfoldensis strain SMR4r
Celeribacter manganoxidans strain DY25
Roseovarius sp AK1035
Sulfitobacter sp SK012
Tateyamaria omphalii strain DOK1 4
Roseobacter denitrificans strain FDAARGOS 309
Roseobacter litoralis Och 149
Antarctobacter heliothermus strain SMS3
Sagittula sp P11
Pelagibaca abyssi strain JLT2014
Thiobacimonas profunda strain JLT2016
Yangia sp CCB
Yangia pacifica strain YSBP01
Marinovum algicola DG 898
Thalassococcus sp SH 1
Ruegeria pomeroyi DSS 3
Ruegeria sp AD91A
Ketogulonicigenium vulgare strain SKV
Ketogulonicigenium robustum strain B003
Sulfitobacter sp D7
Sulfitobacter sp SK025
Sulfitobacter sp AM1 D1
Leisingera methylohalidivorans DSM 14336
Phaeobacter gallaeciensis strain JL2886
Ruegeria sp TM1040
Ruegeria mobilis F1926
Phaeobacter porticola strain P97
Phaeobacter inhibens strain P70
Phaeobacter gallaeciensis strain P63
Phaeobacter piscinae strain P13
Methyloceanibacter caenitepidi Gela4
Rhodobiaceae bacterium strain SMS8
Candidatus Filomicrobium marinum strain W
Hyphomicrobium nitrativorans NL23
Hyphomicrobium sp MC1
Hyphomicrobium denitrificans ATCC 51888
Hyphomicrobium denitrificans 1NES1
Parvibaculum lavamentivorans DS 1
Phreatobacter sp S12
Polymorphum gilvum SL003B 26A1
Pannonibacter phragmitetus BB
Maritalea myrionectae strain HL27085
Pelagibacterium halotolerans B2
Devosia sp H5989
Nitratireductor basaltis strain RR3 28
Hoeflea sp IMCC20628
Shinella sp HZN7
Martelella endophytica strain YC6887
Martelella mediterranea DSM 17316
Martelella sp AD 3
Neorhizobium galegae
Rhizobium sp NT 26
Neorhizobium sp NCHU2750
Rhizobium sp ACO
Agrobacterium vitis S4
Agrobacterium sp RAC06
Agrobacterium tumefaciens strain 1D1108
Agrobacterium fabrum strain 12D13
Agrobacterium tumefaciens strain 1D1609
Agrobacterium rhizogenes strain K599
Rhizobium sp Y9
Agrobacterium tumefaciens strain 12D1
Maricaulis maris MCS10
Parvularcula bermudensis HTCC2503
Brucella canis strain 2009004498
Ochrobactrum pituitosum strain AA2
Ochrobactrum sp A44
Hirschia baltica ATCC 49814
Asticcacaulis excentricus M6
Caulobacter mirabilis strain FWC 38
Caulobacter sp K31
Caulobacter flavus strain RHGG3
Caulobacter segnis strain TK0059
Caulobacter vibrioides strain CB2
Brevundimonas sp LM2
Brevundimonas naejangsanensis strain B1
Brevundimonas naejangsanensis strain BRV3
Brevundimonas sp DS20
Brevundimonas sp GW460
Brevundimonas vesicularis strain FDAARGOS 289
Variibacter gotjawalensis
Breoghania sp LA4
Blastochloris viridis strain ATCC 19567
Blastochloris sp GI
Xanthobacter autotrophicus Py2
Azorhizobium caulinodans ORS 571
Starkeya novella DSM 506
Cellulophaga phage phi10 1
Pseudorhodoplanes sinuspersici strain RIPI110
Oligotropha carboxidovorans OM4
Nitrobacter winogradskyi Nb 255
Nitrobacter hamburgensis X14
Rhodopseudomonas palustris BisB18
Rhodopseudomonas palustris BisA53
Rhodopseudomonas palustris HaA2
Rhodopseudomonas palustris BisB5
Rhodopseudomonas palustris DX 1
Rhodopseudomonas palustris CGA009
Rhodopseudomonas palustris strain YSC3
Pleomorphomonas sp SM30
Chelatococcus sp CO 6
Chelatococcus daeguensis strain TAD1
Hartmannibacter diazotrophicus strain E19T
Methylosinus trichosporium OB3b
Methylocystis sp SC2
Methylocystis rosea strain GW6
Bosea sp Tri 49
Bosea sp AS 1
Bosea sp PAMC 26642
Bosea sp RAC05
Microvirga ossetica strain V5
Microvirga sp 17
Aureimonas sp AU20
Methylobacterium sp 17SD2 17
Methylobacterium radiotolerans JCM 2831
Methylobacterium phyllosphaerae strain CBMB27
Methylobacterium extorquens AM1
Methylobacterium sp AMS5
Methylobacterium populi
Methylobacterium sp DM1

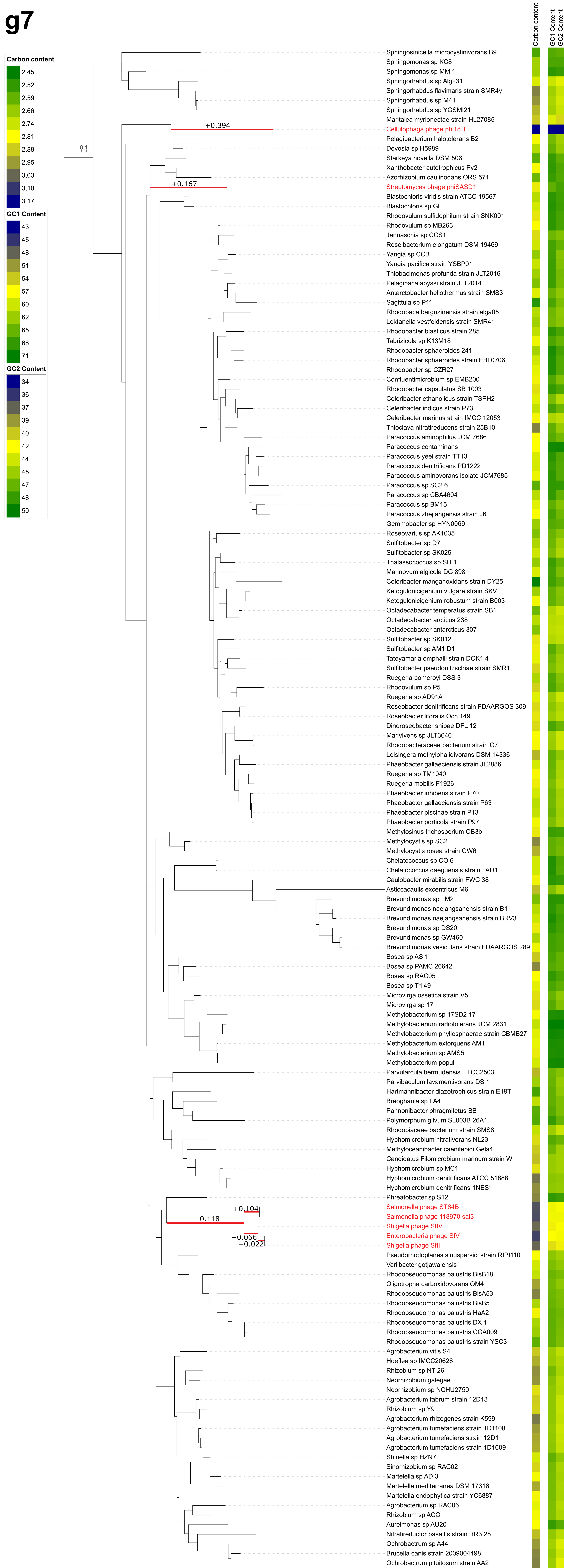
g7
Carbon content
2.45
2.52
2.59
2.66
2.74
2.81
2.88
2.95
3.03
3.10
3.17
GC1 Content
43
45
48
51
54
57
60
62
65
68
71
GC2 Content
34
36
37
39
40
42
44
45
47
48
50
Carbon content
GC1 Content
GC2 Content
0.1
+0.394
+0.167
+0.104
+0.118
+0.066
+0.022
Sphingosinicella microcystinivorans B9
Sphingomonas sp KC8
Sphingomonas sp MM 1
Sphingorhabdus sp Alg231
Sphingorhabdus flavimaris strain SMR4y
Sphingorhabdus sp M41
Sphingorhabdus sp YGSMI21
Maritalea myrionectae strain HL27085
Cellulophaga phage phi18 1
Pelagibacterium halotolerans B2
Devosia sp H5989
Starkeya novella DSM 506
Xanthobacter autotrophicus Py2
Azorhizobium caulinodans ORS 571
Streptomyces phage phiSASD1
Blastochloris viridis strain ATCC 19567
Blastochloris sp GI
Rhodovulum sulfidophilum strain SNK001
Rhodovulum sp MB263
Jannaschia sp CCS1
Roseibacterium elongatum DSM 19469
Yangia sp CCB
Yangia pacifica strain YSBP01
Thiobacimonas profunda strain JLT2016
Pelagibaca abyssi strain JLT2014
Antarctobacter heliothermus strain SMS3
Sagittula sp P11
Rhodobaca barguzinensis strain alga05
Loktanella vestfoldensis strain SMR4r
Rhodobacter blasticus strain 285
Tabrizicola sp K13M18
Rhodobacter sphaeroides 241
Rhodobacter sphaeroides strain EBL0706
Rhodobacter sp CZR27
Confluentimicrobium sp EMB200
Rhodobacter capsulatus SB 1003
Celeribacter ethanolicus strain TSPH2
Celeribacter indicus strain P73
Celeribacter marinus strain IMCC 12053
Thioclava nitratireducens strain 25B10
Paracoccus aminophilus JCM 7686
Paracoccus contaminans
Paracoccus yeei strain TT13
Paracoccus denitrificans PD1222
Paracoccus aminovorans isolate JCM7685
Paracoccus sp SC2 6
Paracoccus sp CBA4604
Paracoccus sp BM15
Paracoccus zhejiangensis strain J6
Gemmobacter sp HYN0069
Roseovarius sp AK1035
Sulfitobacter sp D7
Sulfitobacter sp SK025
Thalassococcus sp SH 1
Marinovum algicola DG 898
Celeribacter manganoxidans strain DY25
Ketogulonicigenium vulgare strain SKV
Ketogulonicigenium robustum strain B003
Octadecabacter temperatus strain SB1
Octadecabacter arcticus 238
Octadecabacter antarcticus 307
Sulfitobacter sp SK012
Sulfitobacter sp AM1 D1
Tateyamaria omphalii strain DOK1 4
Sulfitobacter pseudonitzschiae strain SMR1
Ruegeria pomeroyi DSS 3
Rhodovulum sp P5
Ruegeria sp AD91A
Roseobacter denitrificans strain FDAARGOS 309
Roseobacter litoralis Och 149
Dinoroseobacter shibae DFL 12
Marivivens sp JLT3646
Rhodobacteraceae bacterium strain G7
Leisingera methylohalidivorans DSM 14336
Phaeobacter gallaeciensis strain JL2886
Ruegeria sp TM1040
Ruegeria mobilis F1926
Phaeobacter inhibens strain P70
Phaeobacter gallaeciensis strain P63
Phaeobacter piscinae strain P13
Phaeobacter porticola strain P97
Methylosinus trichosporium OB3b
Methylocystis sp SC2
Methylocystis rosea strain GW6
Chelatococcus sp CO 6
Chelatococcus daeguensis strain TAD1
Caulobacter mirabilis strain FWC 38
Asticcacaulis excentricus M6
Brevundimonas sp LM2
Brevundimonas naejangsanensis strain B1
Brevundimonas naejangsanensis strain BRV3
Brevundimonas sp DS20
Brevundimonas sp GW460
Brevundimonas vesicularis strain FDAARGOS 289
Bosea sp AS 1
Bosea sp PAMC 26642
Bosea sp RAC05
Bosea sp Tri 49
Microvirga ossetica strain V5
Microvirga sp 17
Methylobacterium sp 17SD2 17
Methylobacterium radiotolerans JCM 2831
Methylobacterium phyllosphaerae strain CBMB27
Methylobacterium extorquens AM1
Methylobacterium sp AMS5
Methylobacterium populi
Parvularcula bermudensis HTCC2503
Parvibaculum lavamentivorans DS 1
Hartmannibacter diazotrophicus strain E19T
Breoghania sp LA4
Pannonibacter phragmitetus BB
Polymorphum gilvum SL003B 26A1
Rhodobiaceae bacterium strain SMS8
Hyphomicrobium nitrativorans NL23
Methyloceanibacter caenitepidi Gela4
Candidatus Filomicrobium marinum strain W
Hyphomicrobium sp MC1
Hyphomicrobium denitrificans ATCC 51888
Hyphomicrobium denitrificans 1NES1
Phreatobacter sp S12
Salmonella phage ST64B
Salmonella phage 118970 sal3
Shigella phage SfIV
Enterobacteria phage SfV
Shigella phage SfII
Pseudorhodoplanes sinuspersici strain RIPI110
Variibacter gotjawalensis
Rhodopseudomonas palustris BisB18
Oligotropha carboxidovorans OM4
Rhodopseudomonas palustris BisA53
Rhodopseudomonas palustris BisB5
Rhodopseudomonas palustris HaA2
Rhodopseudomonas palustris DX 1
Rhodopseudomonas palustris CGA009
Rhodopseudomonas palustris strain YSC3
Agrobacterium vitis S4
Hoeflea sp IMCC20628
Rhizobium sp NT 26
Neorhizobium galegae
Neorhizobium sp NCHU2750
Agrobacterium fabrum strain 12D13
Rhizobium sp Y9
Agrobacterium rhizogenes strain K599
Agrobacterium tumefaciens strain 1D1108
Agrobacterium tumefaciens strain 12D1
Agrobacterium tumefaciens strain 1D1609
Shinella sp HZN7
Sinorhizobium sp RAC02
Martelella sp AD 3
Martelella mediterranea DSM 17316
Martelella endophytica strain YC6887
Agrobacterium sp RAC06
Rhizobium sp ACO
Aureimonas sp AU20
Nitratireductor basaltis strain RR3 28
Ochrobactrum sp A44
Brucella canis strain 2009004498
Ochrobactrum pituitosum strain AA2

g10

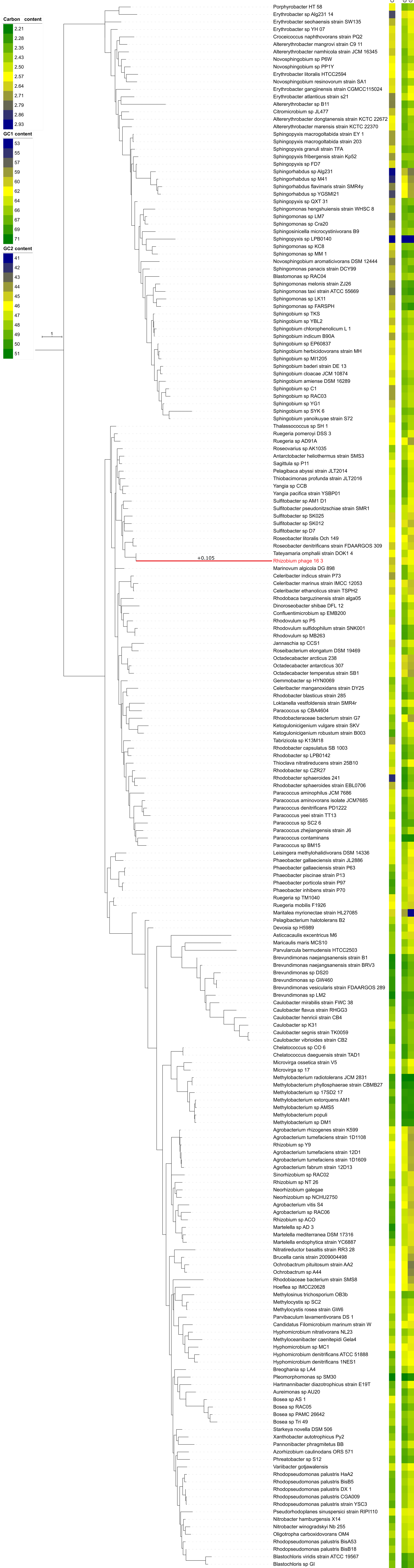
Carbon content
2.21
2.28
2.35
2.43
2.50
2.57
2.64
2.71
2.79
2.86
2.93
GC1 content
53
55
57
59
60
62
64
66
67
69
71
GC2 content
41
42
43
44
45
46
47
48
49
50
51
1
Carbon content
GC1 Content
GC2 Content
+0.105
Porphyrobacter HT 58
Erythrobacter sp Alg231 14
Erythrobacter seohaensis strain SW135
Erythrobacter sp YH 07
Croceicoccus naphthovorans strain PQ2
Altererythrobacter mangrovi strain C9 11
Altererythrobacter namhicola strain JCM 16345
Novosphingobium sp P6W
Novosphingobium sp PP1Y
Erythrobacter litoralis HTCC2594
Novosphingobium resinovorum strain SA1
Erythrobacter gangjinensis strain CGMCC115024
Erythrobacter atlanticus strain s21
Altererythrobacter sp B11
Citromicrobium sp JL477
Altererythrobacter dongtanensis strain KCTC 22672
Altererythrobacter marensis strain KCTC 22370
Sphingopyxis macrogoltabida strain EY 1
Sphingopyxis macrogoltabida strain 203
Sphingopyxis granuli strain TFA
Sphingopyxis fribergensis strain Kp52
Sphingopyxis sp FD7
Sphingorhabdus sp Alg231
Sphingorhabdus sp M41
Sphingorhabdus flavimaris strain SMR4y
Sphingorhabdus sp YGSMI21
Sphingopyxis sp QXT 31
Sphingomonas hengshuiensis strain WHSC 8
Sphingomonas sp LM7
Sphingomonas sp Cra20
Sphingosinicella microcystinivorans B9
Sphingopyxis sp LPB0140
Sphingomonas sp KC8
Sphingomonas sp MM 1
Novosphingobium aromaticivorans DSM 12444
Sphingomonas panacis strain DCY99
Blastomonas sp RAC04
Sphingomonas melonis strain ZJ26
Sphingomonas taxi strain ATCC 55669
Sphingomonas sp LK11
Sphingomonas sp FARSPH
Sphingobium sp TKS
Sphingobium sp YBL2
Sphingobium chlorophenolicum L 1
Sphingobium indicum B90A
Sphingobium sp EP60837
Sphingobium herbicidovorans strain MH
Sphingobium sp MI1205
Sphingobium baderi strain DE 13
Sphingobium cloacae JCM 10874
Sphingobium amiense DSM 16289
Sphingobium sp C1
Sphingobium sp RAC03
Sphingobium sp YG1
Sphingobium sp SYK 6
Sphingobium yanoikuyae strain S72
Thalassococcus sp SH 1
Ruegeria pomeroyi DSS 3
Ruegeria sp AD91A
Roseovarius sp AK1035
Antarctobacter heliothermus strain SMS3
Sagittula sp P11
Pelagibaca abyssi strain JLT2014
Thiobacimonas profunda strain JLT2016
Yangia sp CCB
Yangia pacifica strain YSBP01
Sulfitobacter sp AM1 D1
Sulfitobacter pseudonitzschiae strain SMR1
Sulfitobacter sp SK025
Sulfitobacter sp SK012
Sulfitobacter sp D7
Roseobacter litoralis Och 149
Roseobacter denitrificans strain FDAARGOS 309
Tateyamaria omphalii strain DOK1 4
Rhizobium phage 16 3
Marinovum algicola DG 898
Celeribacter indicus strain P73
Celeribacter marinus strain IMCC 12053
Celeribacter ethanolicus strain TSPH2
Rhodobaca barguzinensis strain alga05
Dinoroseobacter shibae DFL 12
Confluentimicrobium sp EMB200
Rhodovulum sp P5
Rhodovulum sulfidophilum strain SNK001
Rhodovulum sp MB263
Jannaschia sp CCS1
Roseibacterium elongatum DSM 19469
Octadecabacter arcticus 238
Octadecabacter antarcticus 307
Octadecabacter temperatus strain SB1
Gemmobacter sp HYN0069
Celeribacter manganoxidans strain DY25
Rhodobacter blasticus strain 285
Loktanella vestfoldensis strain SMR4r
Paracoccus sp CBA4604
Rhodobacteraceae bacterium strain G7
Ketogulonicigenium vulgare strain SKV
Ketogulonicigenium robustum strain B003
Tabrizicola sp K13M18
Rhodobacter capsulatus SB 1003
Rhodobacter sp LPB0142
Thioclava nitratireducens strain 25B10
Rhodobacter sp CZR27
Rhodobacter sphaeroides 241
Rhodobacter sphaeroides strain EBL0706
Paracoccus aminophilus JCM 7686
Paracoccus aminovorans isolate JCM7685
Paracoccus denitrificans PD1222
Paracoccus yeei strain TT13
Paracoccus sp SC2 6
Paracoccus zhejiangensis strain J6
Paracoccus contaminans
Paracoccus sp BM15
Leisingera methylohalidivorans DSM 14336
Phaeobacter gallaeciensis strain JL2886
Phaeobacter gallaeciensis strain P63
Phaeobacter piscinae strain P13
Phaeobacter porticola strain P97
Phaeobacter inhibens strain P70
Ruegeria sp TM1040
Ruegeria mobilis F1926
Maritalea myrionectae strain HL27085
Pelagibacterium halotolerans B2
Devosia sp H5989
Asticcacaulis excentricus M6
Maricaulis maris MCS10
Parvularcula bermudensis HTCC2503
Brevundimonas naejangsanensis strain B1
Brevundimonas naejangsanensis strain BRV3
Brevundimonas sp DS20
Brevundimonas sp GW460
Brevundimonas vesicularis strain FDAARGOS 289
Brevundimonas sp LM2
Caulobacter mirabilis strain FWC 38
Caulobacter flavus strain RHGG3
Caulobacter henricii strain CB4
Caulobacter sp K31
Caulobacter segnis strain TK0059
Caulobacter vibrioides strain CB2
Chelatococcus sp CO 6
Chelatococcus daeguensis strain TAD1
Microvirga ossetica strain V5
Microvirga sp 17
Methylobacterium radiotolerans JCM 2831
Methylobacterium phyllosphaerae strain CBMB27
Methylobacterium sp 17SD2 17
Methylobacterium extorquens AM1
Methylobacterium sp AMS5
Methylobacterium populi
Methylobacterium sp DM1
Agrobacterium rhizogenes strain K599
Agrobacterium tumefaciens strain 1D1108
Rhizobium sp Y9
Agrobacterium tumefaciens strain 12D1
Agrobacterium tumefaciens strain 1D1609
Agrobacterium fabrum strain 12D13
Sinorhizobium sp RAC02
Rhizobium sp NT 26
Neorhizobium galegae
Neorhizobium sp NCHU2750
Agrobacterium vitis S4
Agrobacterium sp RAC06
Rhizobium sp ACO
Martelella sp AD 3
Martelella mediterranea DSM 17316
Martelella endophytica strain YC6887
Nitratireductor basaltis strain RR3 28
Brucella canis strain 2009004498
Ochrobactrum pituitosum strain AA2
Ochrobactrum sp A44
Rhodobiaceae bacterium strain SMS8
Hoeflea sp IMCC20628
Methylosinus trichosporium OB3b
Methylocystis sp SC2
Methylocystis rosea strain GW6
Parvibaculum lavamentivorans DS 1
Candidatus Filomicrobium marinum strain W
Hyphomicrobium nitrativorans NL23
Methyloceanibacter caenitepidi Gela4
Hyphomicrobium sp MC1
Hyphomicrobium denitrificans ATCC 51888
Hyphomicrobium denitrificans 1NES1
Breoghania sp LA4
Pleomorphomonas sp SM30
Hartmannibacter diazotrophicus strain E19T
Aureimonas sp AU20
Bosea sp AS 1
Bosea sp RAC05
Bosea sp PAMC 26642
Bosea sp Tri 49
Starkeya novella DSM 506
Xanthobacter autotrophicus Py2
Pannonibacter phragmitetus BB
Azorhizobium caulinodans ORS 571
Phreatobacter sp S12
Variibacter gotjawalensis
Rhodopseudomonas palustris HaA2
Rhodopseudomonas palustris BisB5
Rhodopseudomonas palustris DX 1
Rhodopseudomonas palustris CGA009
Rhodopseudomonas palustris strain YSC3
Pseudorhodoplanes sinuspersici strain RIPI110
Nitrobacter hamburgensis X14
Nitrobacter winogradskyi Nb 255
Oligotropha carboxidovorans OM4
Rhodopseudomonas palustris BisA53
Rhodopseudomonas palustris BisB18
Blastochloris viridis strain ATCC 19567
Blastochloris sp GI

g12

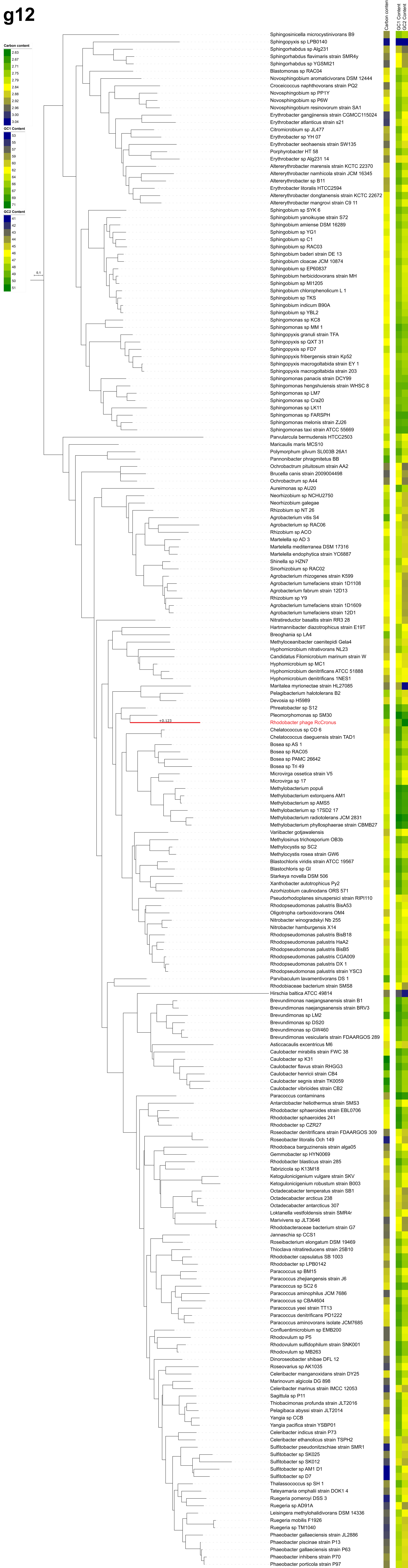

Carbon content
2.63
2.67
2.71
2.75
2.79
2.84
2.88
2.92
2.96
3.00
3.04
GC1 Content
53
55
57
59
60
62
64
66
67
69
71
GC2 Content
41
42
43
44
45
46
47
48
49
50
51
0.1
Carbon content
GC1 Content
GC2 Content
Sphingosinicella microcystinivorans B9
Sphingopyxis sp LPB0140
Sphingorhabdus sp Alg231
Sphingorhabdus flavimaris strain SMR4y
Sphingorhabdus sp YGSMI21
Blastomonas sp RAC04
Novosphingobium aromaticivorans DSM 12444
Croceicoccus naphthovorans strain PQ2
Novosphingobium sp PP1Y
Novosphingobium sp P6W
Novosphingobium resinovorum strain SA1
Erythrobacter gangjinensis strain CGMCC115024
Erythrobacter atlanticus strain s21
Citromicrobium sp JL477
Erythrobacter sp YH 07
Erythrobacter seohaensis strain SW135
Porphyrobacter HT 58
Erythrobacter sp Alg231 14
Altererythrobacter marensis strain KCTC 22370
Altererythrobacter namhicola strain JCM 16345
Altererythrobacter sp B11
Erythrobacter litoralis HTCC2594
Altererythrobacter dongtanensis strain KCTC 22672
Altererythrobacter mangrovi strain C9 11
Sphingobium sp SYK 6
Sphingobium yanoikuyae strain S72
Sphingobium amiense DSM 16289
Sphingobium sp YG1
Sphingobium sp C1
Sphingobium sp RAC03
Sphingobium baderi strain DE 13
Sphingobium cloacae JCM 10874
Sphingobium sp EP60837
Sphingobium herbicidovorans strain MH
Sphingobium sp MI1205
Sphingobium chlorophenolicum L 1
Sphingobium sp TKS
Sphingobium indicum B90A
Sphingobium sp YBL2
Sphingomonas sp KC8
Sphingomonas sp MM 1
Sphingopyxis granuli strain TFA
Sphingopyxis sp QXT 31
Sphingopyxis sp FD7
Sphingopyxis fribergensis strain Kp52
Sphingopyxis macrogoltabida strain EY 1
Sphingopyxis macrogoltabida strain 203
Sphingomonas panacis strain DCY99
Sphingomonas hengshuiensis strain WHSC 8
Sphingomonas sp LM7
Sphingomonas sp Cra20
Sphingomonas sp LK11
Sphingomonas sp FARSPH
Sphingomonas melonis strain ZJ26
Sphingomonas taxi strain ATCC 55669
Parvularcula bermudensis HTCC2503
Maricaulis maris MCS10
Polymorphum gilvum SL003B 26A1
Pannonibacter phragmitetus BB
Ochrobactrum pituitosum strain AA2
Brucella canis strain 2009004498
Ochrobactrum sp A44
Aureimonas sp AU20
Neorhizobium sp NCHU2750
Neorhizobium galegae
Rhizobium sp NT 26
Agrobacterium vitis S4
Agrobacterium sp RAC06
Rhizobium sp ACO
Martelella sp AD 3
Martelella mediterranea DSM 17316
Martelella endophytica strain YC6887
Shinella sp HZN7
Sinorhizobium sp RAC02
Agrobacterium rhizogenes strain K599
Agrobacterium tumefaciens strain 1D1108
Agrobacterium fabrum strain 12D13
Rhizobium sp Y9
Agrobacterium tumefaciens strain 1D1609
Agrobacterium tumefaciens strain 12D1
Nitratireductor basaltis strain RR3 28
Hartmannibacter diazotrophicus strain E19T
Breoghania sp LA4
Methyloceanibacter caenitepidi Gela4
Hyphomicrobium nitrativorans NL23
Candidatus Filomicrobium marinum strain W
Hyphomicrobium sp MC1
Hyphomicrobium denitrificans ATCC 51888
Hyphomicrobium denitrificans 1NES1
Maritalea myrionectae strain HL27085
Pelagibacterium halotolerans B2
Devosia sp H5989
Phreatobacter sp S12
Pleomorphomonas sp SM30
+0.123
Rhodobacter phage RcCronus
Chelatococcus sp CO 6
Chelatococcus daeguensis strain TAD1
Bosea sp AS 1
Bosea sp RAC05
Bosea sp PAMC 26642
Bosea sp Tri 49
Microvirga ossetica strain V5
Microvirga sp 17
Methylobacterium populi
Methylobacterium extorquens AM1
Methylobacterium sp AMS5
Methylobacterium sp 17SD2 17
Methylobacterium radiotolerans JCM 2831
Methylobacterium phyllosphaerae strain CBMB27
Variibacter gotjawalensis
Methylosinus trichosporium OB3b
Methylocystis sp SC2
Methylocystis rosea strain GW6
Blastochloris viridis strain ATCC 19567
Blastochloris sp GI
Starkeya novella DSM 506
Xanthobacter autotrophicus Py2
Azorhizobium caulinodans ORS 571
Pseudorhodoplanes sinuspersici strain RIPI110
Rhodopseudomonas palustris BisA53
Oligotropha carboxidovorans OM4
Nitrobacter winogradskyi Nb 255
Nitrobacter hamburgensis X14
Rhodopseudomonas palustris BisB18
Rhodopseudomonas palustris HaA2
Rhodopseudomonas palustris BisB5
Rhodopseudomonas palustris CGA009
Rhodopseudomonas palustris DX 1
Rhodopseudomonas palustris strain YSC3
Parvibaculum lavamentivorans DS 1
Rhodobiaceae bacterium strain SMS8
Hirschia baltica ATCC 49814
Brevundimonas naejangsanensis strain B1
Brevundimonas naejangsanensis strain BRV3
Brevundimonas sp LM2
Brevundimonas sp DS20
Brevundimonas sp GW460
Brevundimonas vesicularis strain FDAARGOS 289
Asticcacaulis excentricus M6
Caulobacter mirabilis strain FWC 38
Caulobacter sp K31
Caulobacter flavus strain RHGG3
Caulobacter henricii strain CB4
Caulobacter segnis strain TK0059
Caulobacter vibrioides strain CB2
Paracoccus contaminans
Antarctobacter heliothermus strain SMS3
Rhodobacter sphaeroides strain EBL0706
Rhodobacter sphaeroides 241
Rhodobacter sp CZR27
Roseobacter denitrificans strain FDAARGOS 309
Roseobacter litoralis Och 149
Rhodobaca barguzinensis strain alga05
Gemmobacter sp HYN0069
Rhodobacter blasticus strain 285
Tabrizicola sp K13M18
Ketogulonicigenium vulgare strain SKV
Ketogulonicigenium robustum strain B003
Octadecabacter temperatus strain SB1
Octadecabacter arcticus 238
Octadecabacter antarcticus 307
Loktanella vestfoldensis strain SMR4r
Marivivens sp JLT3646
Rhodobacteraceae bacterium strain G7
Jannaschia sp CCS1
Roseibacterium elongatum DSM 19469
Thioclava nitratireducens strain 25B10
Rhodobacter capsulatus SB 1003
Rhodobacter sp LPB0142
Paracoccus sp BM15
Paracoccus zhejiangensis strain J6
Paracoccus sp SC2 6
Paracoccus aminophilus JCM 7686
Paracoccus sp CBA4604
Paracoccus yeei strain TT13
Paracoccus denitrificans PD1222
Paracoccus aminovorans isolate JCM7685
Confluentimicrobium sp EMB200
Rhodovulum sp P5
Rhodovulum sulfidophilum strain SNK001
Rhodovulum sp MB263
Dinoroseobacter shibae DFL 12
Roseovarius sp AK1035
Celeribacter manganoxidans strain DY25
Marinovum algicola DG 898
Celeribacter marinus strain IMCC 12053
Sagittula sp P11
Thiobacimonas profunda strain JLT2016
Pelagibaca abyssi strain JLT2014
Yangia sp CCB
Yangia pacifica strain YSBP01
Celeribacter indicus strain P73
Celeribacter ethanolicus strain TSPH2
Sulfitobacter pseudonitzschiae strain SMR1
Sulfitobacter sp SK025
Sulfitobacter sp SK012
Sulfitobacter sp AM1 D1
Sulfitobacter sp D7
Thalassococcus sp SH 1
Tateyamaria omphalii strain DOK1 4
Ruegeria pomeroyi DSS 3
Ruegeria sp AD91A
Leisingera methylohalidivorans DSM 14336
Ruegeria mobilis F1926
Ruegeria sp TM1040
Phaeobacter gallaeciensis strain JL2886
Phaeobacter piscinae strain P13
Phaeobacter gallaeciensis strain P63
Phaeobacter inhibens strain P70
Phaeobacter porticola strain P97

g13

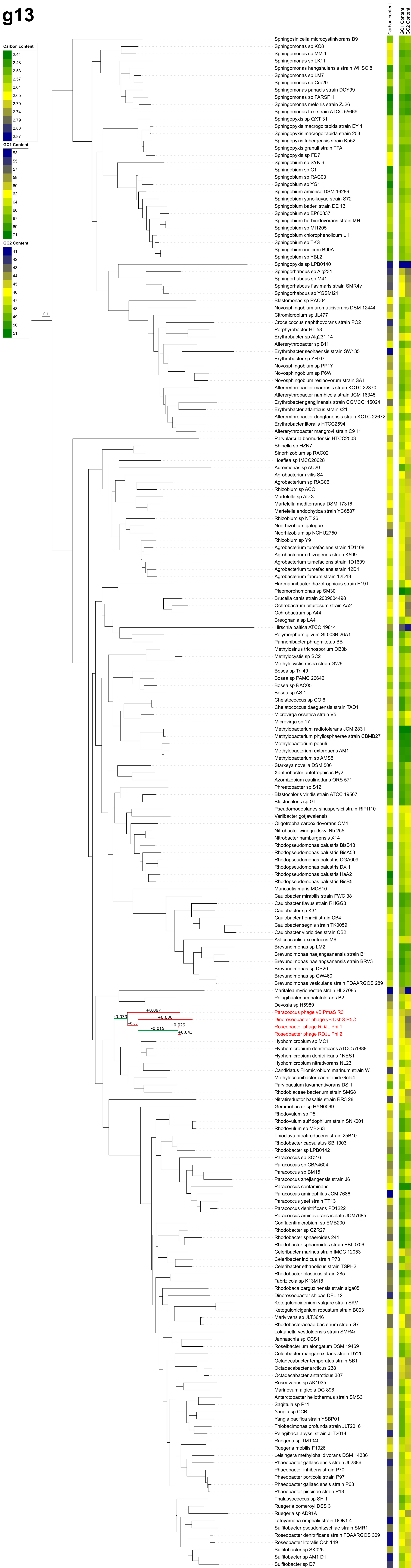

Carbon content
2.44
2.48
2.53
2.57
2.61
2.65
2.70
2.74
2.79
2.83
2.87
GC1 Content
53
55
57
59
60
62
64
66
67
69
71
GC2 Content
41
42
43
44
45
46
47
48
49
50
51
Carbon content
GC1 Content
GC2 Content
0.1
+0.087
+0.036
-0.039
+0.03
-0.015
+0.029
+0.043
Sphingosinicella microcystinivorans B9
Sphingomonas sp KC8
Sphingomonas sp MM 1
Sphingomonas sp LK11
Sphingomonas hengshuiensis strain WHSC 8
Sphingomonas sp LM7
Sphingomonas sp Cra20
Sphingomonas panacis strain DCY99
Sphingomonas sp FARSPH
Sphingomonas melonis strain ZJ26
Sphingomonas taxi strain ATCC 55669
Sphingopyxis sp QXT 31
Sphingopyxis macrogoltabida strain EY 1
Sphingopyxis macrogoltabida strain 203
Sphingopyxis fribergensis strain Kp52
Sphingopyxis granuli strain TFA
Sphingopyxis sp FD7
Sphingobium sp SYK 6
Sphingobium sp C1
Sphingobium sp RAC03
Sphingobium sp YG1
Sphingobium amiense DSM 16289
Sphingobium yanoikuyae strain S72
Sphingobium baderi strain DE 13
Sphingobium sp EP60837
Sphingobium herbicidovorans strain MH
Sphingobium sp MI1205
Sphingobium chlorophenolicum L 1
Sphingobium sp TKS
Sphingobium indicum B90A
Sphingobium sp YBL2
Sphingopyxis sp LPB0140
Sphingorhabdus sp Alg231
Sphingorhabdus sp M41
Sphingorhabdus flavimaris strain SMR4y
Sphingorhabdus sp YGSMI21
Blastomonas sp RAC04
Novosphingobium aromaticivorans DSM 12444
Citromicrobium sp JL477
Croceicoccus naphthovorans strain PQ2
Porphyrobacter HT 58
Erythrobacter sp Alg231 14
Altererythrobacter sp B11
Erythrobacter seohaensis strain SW135
Erythrobacter sp YH 07
Novosphingobium sp PP1Y
Novosphingobium sp P6W
Novosphingobium resinovorum strain SA1
Altererythrobacter marensis strain KCTC 22370
Altererythrobacter namhicola strain JCM 16345
Erythrobacter gangjinensis strain CGMCC115024
Erythrobacter atlanticus strain s21
Altererythrobacter dongtanensis strain KCTC 22672
Erythrobacter litoralis HTCC2594
Altererythrobacter mangrovi strain C9 11
Parvularcula bermudensis HTCC2503
Shinella sp HZN7
Sinorhizobium sp RAC02
Hoeflea sp IMCC20628
Aureimonas sp AU20
Agrobacterium vitis S4
Agrobacterium sp RAC06
Rhizobium sp ACO
Martelella sp AD 3
Martelella mediterranea DSM 17316
Martelella endophytica strain YC6887
Rhizobium sp NT 26
Neorhizobium galegae
Neorhizobium sp NCHU2750
Rhizobium sp Y9
Agrobacterium tumefaciens strain 1D1108
Agrobacterium rhizogenes strain K599
Agrobacterium tumefaciens strain 1D1609
Agrobacterium tumefaciens strain 12D1
Agrobacterium fabrum strain 12D13
Hartmannibacter diazotrophicus strain E19T
Pleomorphomonas sp SM30
Brucella canis strain 2009004498
Ochrobactrum pituitosum strain AA2
Ochrobactrum sp A44
Breoghania sp LA4
Hirschia baltica ATCC 49814
Polymorphum gilvum SL003B 26A1
Pannonibacter phragmitetus BB
Methylosinus trichosporium OB3b
Methylocystis sp SC2
Methylocystis rosea strain GW6
Bosea sp Tri 49
Bosea sp PAMC 26642
Bosea sp RAC05
Bosea sp AS 1
Chelatococcus sp CO 6
Chelatococcus daeguensis strain TAD1
Microvirga ossetica strain V5
Microvirga sp 17
Methylobacterium radiotolerans JCM 2831
Methylobacterium phyllosphaerae strain CBMB27
Methylobacterium populi
Methylobacterium extorquens AM1
Methylobacterium sp AMS5
Starkeya novella DSM 506
Xanthobacter autotrophicus Py2
Azorhizobium caulinodans ORS 571
Phreatobacter sp S12
Blastochloris viridis strain ATCC 19567
Blastochloris sp GI
Pseudorhodoplanes sinuspersici strain RIPI110
Variibacter gotjawalensis
Oligotropha carboxidovorans OM4
Nitrobacter winogradskyi Nb 255
Nitrobacter hamburgensis X14
Rhodopseudomonas palustris BisB18
Rhodopseudomonas palustris BisA53
Rhodopseudomonas palustris CGA009
Rhodopseudomonas palustris DX 1
Rhodopseudomonas palustris HaA2
Rhodopseudomonas palustris BisB5
Maricaulis maris MCS10
Caulobacter mirabilis strain FWC 38
Caulobacter flavus strain RHGG3
Caulobacter sp K31
Caulobacter henricii strain CB4
Caulobacter segnis strain TK0059
Caulobacter vibrioides strain CB2
Asticcacaulis excentricus M6
Brevundimonas sp LM2
Brevundimonas naejangsanensis strain B1
Brevundimonas naejangsanensis strain BRV3
Brevundimonas sp DS20
Brevundimonas sp GW460
Brevundimonas vesicularis strain FDAARGOS 289
Maritalea myrionectae strain HL27085
Pelagibacterium halotolerans B2
Devosia sp H5989
Paracoccus phage vB PmaS R3
Dinoroseobacter phage vB DshS R5C
Roseobacter phage RDJL Phi 1
Roseobacter phage RDJL Phi 2
Hyphomicrobium sp MC1
Hyphomicrobium denitrificans ATCC 51888
Hyphomicrobium denitrificans 1NES1
Hyphomicrobium nitrativorans NL23
Candidatus Filomicrobium marinum strain W
Methyloceanibacter caenitepidi Gela4
Parvibaculum lavamentivorans DS 1
Rhodobiaceae bacterium strain SMS8
Nitratireductor basaltis strain RR3 28
Gemmobacter sp HYN0069
Rhodovulum sp P5
Rhodovulum sulfidophilum strain SNK001
Rhodovulum sp MB263
Thioclava nitratireducens strain 25B10
Rhodobacter capsulatus SB 1003
Rhodobacter sp LPB0142
Paracoccus sp SC2 6
Paracoccus sp CBA4604
Paracoccus sp BM15
Paracoccus zhejiangensis strain J6
Paracoccus contaminans
Paracoccus aminophilus JCM 7686
Paracoccus yeei strain TT13
Paracoccus denitrificans PD1222
Paracoccus aminovorans isolate JCM7685
Confluentimicrobium sp EMB200
Rhodobacter sp CZR27
Rhodobacter sphaeroides 241
Rhodobacter sphaeroides strain EBL0706
Celeribacter marinus strain IMCC 12053
Celeribacter indicus strain P73
Celeribacter ethanolicus strain TSPH2
Rhodobacter blasticus strain 285
Tabrizicola sp K13M18
Rhodobaca barguzinensis strain alga05
Dinoroseobacter shibae DFL 12
Ketogulonicigenium vulgare strain SKV
Ketogulonicigenium robustum strain B003
Marivivens sp JLT3646
Rhodobacteraceae bacterium strain G7
Loktanella vestfoldensis strain SMR4r
Jannaschia sp CCS1
Roseibacterium elongatum DSM 19469
Celeribacter manganoxidans strain DY25
Octadecabacter temperatus strain SB1
Octadecabacter arcticus 238
Octadecabacter antarcticus 307
Roseovarius sp AK1035
Marinovum algicola DG 898
Antarctobacter heliothermus strain SMS3
Sagittula sp P11
Yangia sp CCB
Yangia pacifica strain YSBP01
Thiobacimonas profunda strain JLT2016
Pelagibaca abyssi strain JLT2014
Ruegeria sp TM1040
Ruegeria mobilis F1926
Leisingera methylohalidivorans DSM 14336
Phaeobacter gallaeciensis strain JL2886
Phaeobacter inhibens strain P70
Phaeobacter porticola strain P97
Phaeobacter gallaeciensis strain P63
Phaeobacter piscinae strain P13
Thalassococcus sp SH 1
Ruegeria pomeroyi DSS 3
Ruegeria sp AD91A
Tateyamaria omphalii strain DOK1 4
Sulfitobacter pseudonitzschiae strain SMR1
Roseobacter denitrificans strain FDAARGOS 309
Roseobacter litoralis Och 149
Sulfitobacter sp SK025
Sulfitobacter sp AM1 D1
Sulfitobacter sp D7

g14

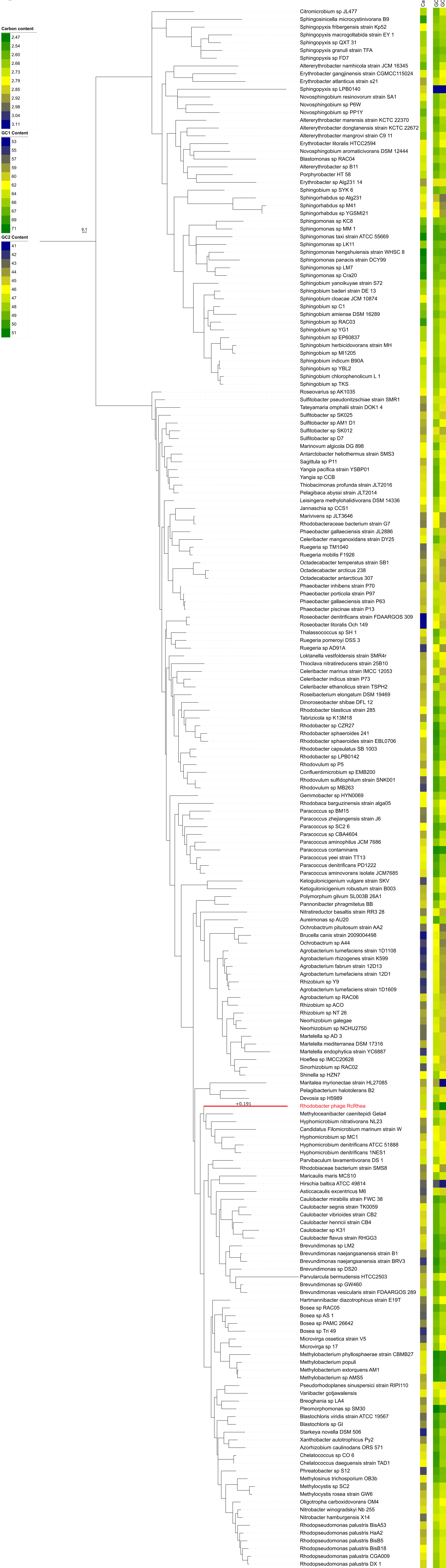

Carbon content
2.47
2.54
2.60
2.66
2.73
2.79
2.85
2.92
2.98
3.04
3.11
GC1 Content
53
55
57
59
60
62
64
66
67
69
71
GC2 Content
41
42
43
44
45
46
47
48
49
50
51
0.1
Carbon content
GC1 Content
GC2 Content
Citromicrobium sp JL477
Sphingosinicella microcystinivorans B9
Sphingopyxis fribergensis strain Kp52
Sphingopyxis macrogoltabida strain EY 1
Sphingopyxis sp QXT 31
Sphingopyxis granuli strain TFA
Sphingopyxis sp FD7
Altererythrobacter namhicola strain JCM 16345
Erythrobacter gangjinensis strain CGMCC115024
Erythrobacter atlanticus strain s21
Sphingopyxis sp LPB0140
Novosphingobium resinovorum strain SA1
Novosphingobium sp P6W
Novosphingobium sp PP1Y
Altererythrobacter marensis strain KCTC 22370
Altererythrobacter dongtanensis strain KCTC 22672
Altererythrobacter mangrovi strain C9 11
Erythrobacter litoralis HTCC2594
Novosphingobium aromaticivorans DSM 12444
Blastomonas sp RAC04
Altererythrobacter sp B11
Porphyrobacter HT 58
Erythrobacter sp Alg231 14
Sphingobium sp SYK 6
Sphingorhabdus sp Alg231
Sphingorhabdus sp M41
Sphingorhabdus sp YGSMI21
Sphingomonas sp KC8
Sphingomonas sp MM 1
Sphingomonas taxi strain ATCC 55669
Sphingomonas sp LK11
Sphingomonas hengshuiensis strain WHSC 8
Sphingomonas panacis strain DCY99
Sphingomonas sp LM7
Sphingomonas sp Cra20
Sphingobium yanoikuyae strain S72
Sphingobium baderi strain DE 13
Sphingobium cloacae JCM 10874
Sphingobium sp C1
Sphingobium amiense DSM 16289
Sphingobium sp RAC03
Sphingobium sp YG1
Sphingobium sp EP60837
Sphingobium herbicidovorans strain MH
Sphingobium sp MI1205
Sphingobium indicum B90A
Sphingobium sp YBL2
Sphingobium chlorophenolicum L 1
Sphingobium sp TKS
Roseovarius sp AK1035
Sulfitobacter pseudonitzschiae strain SMR1
Tateyamaria omphalii strain DOK1 4
Sulfitobacter sp SK025
Sulfitobacter sp AM1 D1
Sulfitobacter sp SK012
Sulfitobacter sp D7
Marinovum algicola DG 898
Antarctobacter heliothermus strain SMS3
Sagittula sp P11
Yangia pacifica strain YSBP01
Yangia sp CCB
Thiobacimonas profunda strain JLT2016
Pelagibaca abyssi strain JLT2014
Leisingera methylohalidivorans DSM 14336
Jannaschia sp CCS1
Marivivens sp JLT3646
Rhodobacteraceae bacterium strain G7
Phaeobacter gallaeciensis strain JL2886
Celeribacter manganoxidans strain DY25
Ruegeria sp TM1040
Ruegeria mobilis F1926
Octadecabacter temperatus strain SB1
Octadecabacter arcticus 238
Octadecabacter antarcticus 307
Phaeobacter inhibens strain P70
Phaeobacter porticola strain P97
Phaeobacter gallaeciensis strain P63
Phaeobacter piscinae strain P13
Roseobacter denitrificans strain FDAARGOS 309
Roseobacter litoralis Och 149
Thalassococcus sp SH 1
Ruegeria pomeroyi DSS 3
Ruegeria sp AD91A
Loktanella vestfoldensis strain SMR4r
Thioclava nitratireducens strain 25B10
Celeribacter marinus strain IMCC 12053
Celeribacter indicus strain P73
Celeribacter ethanolicus strain TSPH2
Roseibacterium elongatum DSM 19469
Dinoroseobacter shibae DFL 12
Rhodobacter blasticus strain 285
Tabrizicola sp K13M18
Rhodobacter sp CZR27
Rhodobacter sphaeroides 241
Rhodobacter sphaeroides strain EBL0706
Rhodobacter capsulatus SB 1003
Rhodobacter sp LPB0142
Rhodovulum sp P5
Confluentimicrobium sp EMB200
Rhodovulum sulfidophilum strain SNK001
Rhodovulum sp MB263
Gemmobacter sp HYN0069
Rhodobaca barguzinensis strain alga05
Paracoccus sp BM15
Paracoccus zhejiangensis strain J6
Paracoccus sp SC2 6
Paracoccus sp CBA4604
Paracoccus aminophilus JCM 7686
Paracoccus contaminans
Paracoccus yeei strain TT13
Paracoccus denitrificans PD1222
Paracoccus aminovorans isolate JCM7685
Ketogulonicigenium vulgare strain SKV
Ketogulonicigenium robustum strain B003
Polymorphum gilvum SL003B 26A1
Pannonibacter phragmitetus BB
Nitratireductor basaltis strain RR3 28
Aureimonas sp AU20
Ochrobactrum pituitosum strain AA2
Brucella canis strain 2009004498
Ochrobactrum sp A44
Agrobacterium tumefaciens strain 1D1108
Agrobacterium rhizogenes strain K599
Agrobacterium fabrum strain 12D13
Agrobacterium tumefaciens strain 12D1
Rhizobium sp Y9
Agrobacterium tumefaciens strain 1D1609
Agrobacterium sp RAC06
Rhizobium sp ACO
Rhizobium sp NT 26
Neorhizobium galegae
Neorhizobium sp NCHU2750
Martelella sp AD 3
Martelella mediterranea DSM 17316
Martelella endophytica strain YC6887
Hoeflea sp IMCC20628
Sinorhizobium sp RAC02
Shinella sp HZN7
Maritalea myrionectae strain HL27085
Pelagibacterium halotolerans B2
Devosia sp H5989
+0.191
Rhodobacter phage RcRhea
Methyloceanibacter caenitepidi Gela4
Hyphomicrobium nitrativorans NL23
Candidatus Filomicrobium marinum strain W
Hyphomicrobium sp MC1
Hyphomicrobium denitrificans ATCC 51888
Hyphomicrobium denitrificans 1NES1
Parvibaculum lavamentivorans DS 1
Rhodobiaceae bacterium strain SM58
Maricaulis maris MCS10
Hirschia baltica ATCC 49814
Asticcacaulis excentricus M6
Caulobacter mirabilis strain FWC 38
Caulobacter segnis strain TK0059
Caulobacter vibrioides strain CB2
Caulobacter henricii strain CB4
Caulobacter sp K31
Caulobacter flavus strain RHGG3
Brevundimonas sp LM2
Brevundimonas naejangsanensis strain B1
Brevundimonas naejangsanensis strain BRV3
Brevundimonas sp DS20
Parvularcula bermudensis HTCC2503
Brevundimonas sp GW460
Brevundimonas vesicularis strain FDAARGOS 289
Hartmannibacter diazotrophicus strain E19T
Bosea sp RAC05
Bosea sp AS 1
Bosea sp PAMC 26642
Bosea sp Tri 49
Microvirga ossetica strain V5
Microvirga sp 17
Methylobacterium phyllosphaerae strain CBMB27
Methylobacterium populi
Methylobacterium extorquens AM1
Methylobacterium sp AMS5
Pseudorhodoplanes sinuspersici strain RIPI110
Variibacter gotjawalensis
Breoghania sp LA4
Pleomorphomonas sp SM30
Blastochloris viridis strain ATCC 19567
Blastochloris sp GI
Starkeya novella DSM 506
Xanthobacter autotrophicus Py2
Azorhizobium caulinodans ORS 571
Chelatococcus sp CO 6
Chelatococcus daeguensis strain TAD1
Phreatobacter sp S12
Methylosinus trichosporium OB3b
Methylocystis sp SC2
Methylocystis rosea strain GW6
Oligotropha carboxidovorans OM4
Nitrobacter winogradskyi Nb 255
Nitrobacter hamburgensis X14
Rhodopseudomonas palustris BisA53
Rhodopseudomonas palustris HaA2
Rhodopseudomonas palustris BisB5
Rhodopseudomonas palustris BisB18
Rhodopseudomonas palustris CGA009
Rhodopseudomonas palustris DX 1

g15

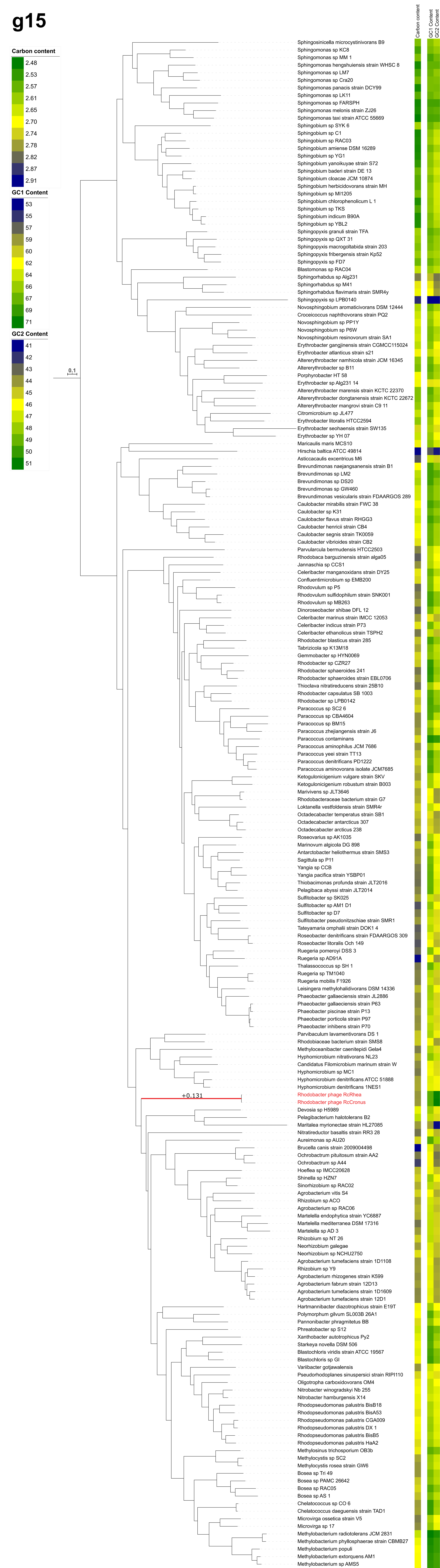
Carbon content
2.48
2.53
2.57
2.61
2.65
2.70
2.74
2.78
2.82
2.87
2.91
GC1 Content
53
55
57
59
60
62
64
66
67
69
71
GC2 Content
41
42
43
44
45
46
47
48
49
50
51
0.1
+0.131
Carbon content
GC1 Content
GC2 Content
Sphingosinicella microcystinivorans B9
Sphingomonas sp KC8
Sphingomonas sp MM 1
Sphingomonas hengshuiensis strain WHSC 8
Sphingomonas sp LM7
Sphingomonas sp Cra20
Sphingomonas panacis strain DCY99
Sphingomonas sp LK11
Sphingomonas sp FARSPH
Sphingomonas melonis strain ZJ26
Sphingomonas taxi strain ATCC 55669
Sphingobium sp SYK 6
Sphingobium sp C1
Sphingobium sp RAC03
Sphingobium amiense DSM 16289
Sphingobium sp YG1
Sphingobium yanoikuyae strain S72
Sphingobium baderi strain DE 13
Sphingobium cloacae JCM 10874
Sphingobium herbicidovorans strain MH
Sphingobium sp MI1205
Sphingobium chlorophenolicum L 1
Sphingobium sp TKS
Sphingobium indicum B90A
Sphingobium sp YBL2
Sphingopyxis granuli strain TFA
Sphingopyxis sp QXT 31
Sphingopyxis macrogoltabida strain 203
Sphingopyxis fribergensis strain Kp52
Sphingopyxis sp FD7
Blastomonas sp RAC04
Sphingorhabdus sp Alg231
Sphingorhabdus sp M41
Sphingorhabdus flavimaris strain SMR4y
Sphingopyxis sp LPB0140
Novosphingobium aromaticivorans DSM 12444
Croceicoccus naphthovorans strain PQ2
Novosphingobium sp PP1Y
Novosphingobium sp P6W
Novosphingobium resinovorum strain SA1
Erythrobacter gangjinensis strain CGMCC115024
Erythrobacter atlanticus strain s21
Altererythrobacter namhicola strain JCM 16345
Altererythrobacter sp B11
Porphyrobacter HT 58
Erythrobacter sp Alg231 14
Altererythrobacter marensis strain KCTC 22370
Altererythrobacter dongtanensis strain KCTC 22672
Altererythrobacter mangrovi strain C9 11
Citromicrobium sp JL477
Erythrobacter litoralis HTCC2594
Erythrobacter seohaensis strain SW135
Erythrobacter sp YH 07
Maricaulis maris MCS10
Hirschia baltica ATCC 49814
Asticcacaulis excentricus M6
Brevundimonas naejangsanensis strain B1
Brevundimonas sp LM2
Brevundimonas sp DS20
Brevundimonas sp GW460
Brevundimonas vesicularis strain FDAARGOS 289
Caulobacter mirabilis strain FWC 38
Caulobacter sp K31
Caulobacter flavus strain RHGG3
Caulobacter henricii strain CB4
Caulobacter segnis strain TK0059
Caulobacter vibrioides strain CB2
Parvularcula bermudensis HTCC2503
Rhodobaca barguzinensis strain alga05
Jannaschia sp CCS1
Celeribacter manganoxidans strain DY25
Confluentimicrobium sp EMB200
Rhodovulum sp P5
Rhodovulum sulfidophilum strain SNK001
Rhodovulum sp MB263
Dinoroseobacter shibae DFL 12
Celeribacter marinus strain IMCC 12053
Celeribacter indicus strain P73
Celeribacter ethanolicus strain TSPH2
Rhodobacter blasticus strain 285
Tabrizicola sp K13M18
Gemmobacter sp HYN0069
Rhodobacter sp CZR27
Rhodobacter sphaeroides 241
Rhodobacter sphaeroides strain EBL0706
Thioclava nitratireducens strain 25B10
Rhodobacter capsulatus SB 1003
Rhodobacter sp LPB0142
Paracoccus sp SC2 6
Paracoccus sp CBA4604
Paracoccus sp BM15
Paracoccus zhejiangensis strain J6
Paracoccus contaminans
Paracoccus aminophilus JCM 7686
Paracoccus yeei strain TT13
Paracoccus denitrificans PD1222
Paracoccus aminovorans isolate JCM7685
Ketogulonicigenium vulgare strain SKV
Ketogulonicigenium robustum strain B003
Marivivens sp JLT3646
Rhodobacteraceae bacterium strain G7
Loktanella vestfoldensis strain SMR4r
Octadecabacter temperatus strain SB1
Octadecabacter antarcticus 307
Octadecabacter arcticus 238
Roseovarius sp AK1035
Marinovum algicola DG 898
Antarctobacter heliothermus strain SMS3
Sagittula sp P11
Yangia sp CCB
Yangia pacifica strain YSBP01
Thiobacimonas profunda strain JLT2016
Pelagibaca abyssi strain JLT2014
Sulfitobacter sp SK025
Sulfitobacter sp AM1 D1
Sulfitobacter sp D7
Sulfitobacter pseudonitzschiae strain SMR1
Tateyamaria omphalii strain DOK1 4
Roseobacter denitrificans strain FDAARGOS 309
Roseobacter litoralis Och 149
Ruegeria pomeroyi DSS 3
Ruegeria sp AD91A
Thalassococcus sp SH 1
Ruegeria sp TM1040
Ruegeria mobilis F1926
Leisingera methylohalidivorans DSM 14336
Phaeobacter gallaeciensis strain JL2886
Phaeobacter gallaeciensis strain P63
Phaeobacter piscinae strain P13
Phaeobacter porticola strain P97
Phaeobacter inhibens strain P70
Parvibaculum lavamentivorans DS 1
Rhodobiaceae bacterium strain SMS8
Methyloceanibacter caenitepidi Gela4
Hyphomicrobium nitrativorans NL23
Candidatus Filomicrobium marinum strain W
Hyphomicrobium sp MC1
Hyphomicrobium denitrificans ATCC 51888
Hyphomicrobium denitrificans 1NES1
Rhodobacter phage RcRhea
Rhodobacter phage RcCronus
Devosia sp H5989
Pelagibacterium halotolerans B2
Maritalea myrionectae strain HL27085
Nitratireductor basaltis strain RR3 28
Aureimonas sp AU20
Brucella canis strain 2009004498
Ochrobactrum pituitosum strain AA2
Ochrobactrum sp A44
Hoeflea sp IMCC20628
Shinella sp HZN7
Sinorhizobium sp RAC02
Agrobacterium vitis S4
Rhizobium sp ACO
Agrobacterium sp RAC06
Martelella endophytica strain YC6887
Martelella mediterranea DSM 17316
Martelella sp AD 3
Rhizobium sp NT 26
Neorhizobium galegae
Neorhizobium sp NCHU2750
Agrobacterium tumefaciens strain 1D1108
Rhizobium sp Y9
Agrobacterium rhizogenes strain K599
Agrobacterium fabrum strain 12D13
Agrobacterium tumefaciens strain 1D1609
Agrobacterium tumefaciens strain 12D1
Hartmannibacter diazotrophicus strain E19T
Polymorphum gilvum SL003B 26A1
Pannonibacter phragmitetus BB
Phreatobacter sp S12
Xanthobacter autotrophicus Py2
Starkeya novella DSM 506
Blastochloris viridis strain ATCC 19567
Blastochloris sp GI
Variibacter gotjawalensis
Pseudorhodoplanes sinuspersici strain RIPI110
Oligotropha carboxidovorans OM4
Nitrobacter winogradskyi Nb 255
Nitrobacter hamburgensis X14
Rhodopseudomonas palustris BisB18
Rhodopseudomonas palustris BisA53
Rhodopseudomonas palustris CGA009
Rhodopseudomonas palustris DX 1
Rhodopseudomonas palustris BisB5
Rhodopseudomonas palustris HaA2
Methylosinus trichosporium OB3b
Methylocystis sp SC2
Methylocystis rosea strain GW6
Bosea sp Tri 49
Bosea sp PAMC 26642
Bosea sp RAC05
Bosea sp AS 1
Chelatococcus sp CO 6
Chelatococcus daeguensis strain TAD1
Microvirga ossetica strain V5
Microvirga sp 17
Methylobacterium radiotolerans JCM 2831
Methylobacterium phyllosphaerae strain CBMB27
Methylobacterium populi
Methylobacterium extorquens AM1
Methylobacterium sp AMS5
