# Supplementary material for: Selection for Reducing Energy Cost of Protein Production Drives the GC Content and Amino Acid Composition Bias in Gene Transfer Agents
Source: mBio. 2020 Jul 14;11(4):e01206-20. doi: 10.1128/mBio.01206-20 (PMC7360931; doi:10.1128/mBio.01206-20)
Supplement: TABLE S3 [file mBio.01206-20-st003.pdf]

Table S3. The likelihood ratio test for the branch site A model.

GTA gene	LRT	p-value	Corrected p-value
g2$	**52.23**	**4.94E-13**	**6.91E-12**
g3	**58.29**	**2.26E-14**	**3.17E-13**
g4	**28.52**	**9.27E-08**	**1.30E-06**
g5	**33.89**	**5.83E-09**	**8.16E-08**
g6	**19.49**	**1.01E-05**	**0.00014112**
g7	11.34	0.000759	0.0106204
g8	1.14	0.2857	1
g9	**14.98**	**0.000109**	**0.0015218**
g10	9.16	0.002474	0.034636
g11	6.09	0.01359	0.19026
*g12**	**49.76**	**1.74E-12**	**2.43E-11**
g13	**51.84**	**6.02E-13**	**8.43E-12**
g14	5.4	0.02014	0.28196
*g15**	**153.36**	**3.20E-35**	**4.47E-34**

*To reduce computation time, for *g12* and *g15* genes the alignments were trimmed by removing sites that had > 50% and >20% gaps, respectively.

$Genes in bold font have corrected p-values < 0.01
