# Supplementary material for: Selection for Reducing Energy Cost of Protein Production Drives the GC Content and Amino Acid Composition Bias in Gene Transfer Agents
Source: mBio. 2020 Jul 14;11(4):e01206-20. doi: 10.1128/mBio.01206-20 (PMC7360931; doi:10.1128/mBio.01206-20)
Supplement: TABLE S4 [file mBio.01206-20-st004.pdf]

Table S4. The average number of carbons per amino acid side chain in *Sphigomonadales* and three other orders combined together.

GTA gene	*Sphingomonadales* average	Other orders average	p-value	Corrected p-value
g2[$]	**2.7**	**2.79**	**6.70E-17**	**9.38E-16**
g3	**2.71**	**2.81**	**3.80E-16**	**5.32E-15**
g4	**2.73**	**2.8**	**0.00017**	**0.00238**
g5	**2.65**	**2.75**	**1.80E-21**	**2.52E-20**
g6	**2.72**	**2.82**	**2.20E-09**	**3.08E-08**
g7	2.79	2.79	0.47	1
g8	2.65	2.71	0.99	1
g9	**2.53**	**2.67**	**4.40E-22**	**6.16E-21**
g10	2.67	2.46	1	1
g11	2.34	2.37	0.004	0.056
g12[*]	**2.74**	**2.86**	**5.10E-15**	**7.14E-14**
g13	2.63	2.68	0.003	0.042
g14	2.69	2.89	7.80E-19	1.09E-17
g15[*]	**2.62**	**2.71**	**2.96E-11**	**4.15E-10**

[*]For g12 and g15 genes the alignments were trimmed by removing sites that had >50% and >20% gaps, respectively.

[$]Genes in bold font have sites under the positive selections and corrected p-values < 0.01
