# Supplementary material for: Selection for Reducing Energy Cost of Protein Production Drives the GC Content and Amino Acid Composition Bias in Gene Transfer Agents
Source: mBio. 2020 Jul 14;11(4):e01206-20. doi: 10.1128/mBio.01206-20 (PMC7360931; doi:10.1128/mBio.01206-20)
Supplement: TABLE S5 [file mBio.01206-20-st005.pdf]

**Table S5. Contribution of sites under the positive selection to the carbon utilization in *Sphingomonadales'* GTA genes.**

| Site | Bayes empirical Bayes (probability) | Average change in number of carbons | p-value |
|---|---|---|---|
| | | **g2** | |
| **361** | 1 | +6.00 | 1.0000 |
| **433** | 1 | 0.00 | 1.0000 |
| **466** | 0.95 | +0.26 | 0.9856 |
| 479[$] | **1** | **-2.20** | **0.0000** |
| 503 | **1** | **-0.93** | **0.0000** |
| **505** | 1 | +0.10 | 0.9940 |
| 506 | **1** | **-3.39** | **0.0000** |
| 683 | **0.98** | **-1.16** | **0.0000** |
| 759 | **0.99** | **-1.31** | **0.0000** |
| **762** | 0.99 | +0.18 | 0.8826 |
| 812 | **0.98** | **-2.33** | **0.0000** |
| **815** | 1 | +1.90 | 1.0000 |
| **821** | 0.97 | 0.00 | 1.0000 |
| **Site** | **Bayes empirical Bayes (probability)** | **Average change in number of carbons** | **p-value** |
| | | **g3** | |
| 127 | **0.962** | **-0.74** | **0.0000** |
| **140** | 0.974 | +3.26 | 1.0000 |
| 142 | **0.95** | **-1.29** | **0.0000** |
| **188** | 0.985 | +0.36 | 0.9127 |
| 193 | **0.988** | **-1.55** | **0.0000** |
| **199** | 0.964 | +0.92 | 1.0000 |
| 205 | **0.995** | **-2.98** | **0.0000** |
| 211 | **0.969** | **-0.72** | **0.0000** |
| 216 | **0.952** | **-0.66** | **0.0000** |
| 245 | **0.971** | **-0.75** | **0.0000** |
| 247 | **0.995** | **-1.70** | **0.0000** |
| 248 | **0.972** | **-0.89** | **0.0000** |
| 249 | **0.984** | **-0.88** | **0.0000** |
| 256 | **0.992** | **-1.34** | **0.0000** |
| 262 | **0.999** | **-5.53** | **0.0000** |
| **268** | 0.993 | +2.82 | 1.0000 |
| **274** | 0.967 | +0.06 | 0.1559 |
| **349** | 0.973 | +2.75 | 1.0000 |
| 375 | **0.971** | **-2.22** | **0.0000** |
| 376 | **0.961** | **-1.08** | **0.0000** |
| 386 | **0.955** | **-0.96** | **0.0000** |

| | | | |
|---|---|---|---|
| 396 | **0.97** | **-0.36** | **0.0000** |
| **400** | 0.952 | +0.19 | 1.0000 |
| 438 | **0.953** | **-1.23** | **0.0000** |
| **454** | 0.993 | +1.03 | 1.0000 |
| 456 | **0.98** | **-0.68** | **0.0000** |
| 469 | **0.968** | **-1.84** | **0.0000** |
| **491** | 0.989 | +0.02 | 0.1556 |
| 507 | **0.975** | **-2.65** | **0.0000** |
| **520** | 0.954 | +2.31 | 1.0000 |
| 526 | **0.973** | **-8.57** | **0.0000** |
| 572 | **0.992** | **-0.34** | **0.0183** |
| **586** | 0.979 | +1.57 | 1.0000 |
| **Site** | **Bayes empirical Bayes (probability)** | **Average change in number of carbons** | **p-value** |
| | **g4** | | |
| **110** | 0.969 | +1.52 | 0.9999 |
| **115** | 1 | 0.00 | 1.0000 |
| 116 | **0.958** | **-0.26** | **0.0000** |
| **122** | 0.964 | -0.35 | 0.6569 |
| **128** | 0.95 | +1.34 | 1.0000 |
| **135** | 0.951 | +0.71 | 1.0000 |
| **151** | 0.971 | +0.26 | 0.9976 |
| 152 | **0.989** | **-0.88** | **0.0000** |
| **153** | 0.986 | +0.91 | 1.0000 |
| **165** | 0.951 | +1.07 | 0.7478 |
| 166 | **1** | **-5.50** | **0.0000** |
| 169 | **0.961** | **-0.87** | **0.0000** |
| 177 | **1** | **-2.76** | **0.0000** |
| **179** | 0.97 | +0.09 | 0.6501 |
| 183 | **0.99** | **-1.52** | **0.0000** |
| 184 | **0.951** | **-0.57** | **0.0006** |
| **185** | 0.952 | +0.70 | 1.0000 |
| 186 | **0.974** | **-0.72** | **0.0000** |
| 188 | **0.994** | **-1.82** | **0.0000** |
| **196** | 0.989 | +0.30 | 0.9984 |
| **206** | 0.993 | +2.71 | 1.0000 |
| **208** | 0.994 | 0.00 | 1.0000 |
| **210** | 0.999 | +0.48 | 1.0000 |
| **211** | 0.993 | +0.53 | 1.0000 |
| 227 | **0.976** | **-1.86** | **0.0000** |
| 238 | **0.984** | **-0.66** | **0.0000** |
| 245 | **1** | **-5.29** | **0.0000** |
| 255 | **0.951** | **-0.77** | **0.0000** |

| | | | |
|---|---|---|---|
| **257** | 0.999 | +1.16 | 1.0000 |
| **Site** | **Bayes empirical Bayes (probability)** | **Average change in number of carbons** | **p-value** |
| | **g5** | | |
| **413** | 0.981 | +5.22 | 1.0000 |
| 414 | **0.976** | **-3.44** | **0.0000** |
| 450 | **0.954** | **-2.88** | **0.0000** |
| 524 | **0.986** | **-1.08** | **0.0000** |
| 586 | **0.992** | **-1.40** | **0.0000** |
| 588 | **0.952** | **-1.04** | **0.0000** |
| **590** | 0.988 | +2.99 | 1.0000 |
| 614 | **0.982** | **-3.43** | **0.0000** |
| 623 | **0.98** | **-1.93** | **0.0000** |
| **635** | 0.973 | +1.15 | 1.0000 |
| **710** | 0.976 | +1.41 | 1.0000 |
| 712 | **0.977** | **-0.27** | **0.0000** |
| **Site** | **Bayes empirical Bayes (probability)** | **Average change in number of carbons** | **p-value** |
| | **g6** | | |
| 103 | **0.979** | **-2.51** | **0.0000** |
| 118 | **0.971** | **-2.21** | **0.0000** |
| **178** | 0.956 | -0.06 | 0.1520 |
| 188 | **0.991** | **-0.66** | **0.0000** |
| **192** | 0.98 | +0.41 | 1.0000 |
| **227** | 0.953 | +1.11 | 1.0000 |
| 231 | **0.986** | **-2.57** | **0.0000** |
| 281 | **0.991** | **-0.43** | **0.0000** |
| **289** | 0.999 | +5.36 | 1.0000 |
| **290** | 0.968 | +1.99 | 1.0000 |
| **292** | 0.969 | +1.35 | 1.0000 |
| **Site** | **Bayes empirical Bayes (probability)** | **Average change in number of carbons** | **p-value** |
| | **g9** | | |
| 7 | **1** | **-2.93** | **0.0000** |
| 8 | **0.992** | **-0.97** | **0.0000** |
| **27** | 0.993 | +0.03 | 0.6380 |
| 36 | **0.962** | **-1.11** | **0.0000** |
| 38 | **0.995** | **-0.39** | **0.0000** |
| 40 | **0.982** | **-3.00** | **0.0000** |
| 42 | **0.99** | **-1.01** | **0.0000** |
| **46** | 0.992 | +0.33 | 1.0000 |
| **50** | 1 | +1.81 | 1.0000 |
| 65 | **0.986** | **-0.24** | **0.0000** |

| | | | |
|---|---|---|---|
| 67 | **1** | **-1.19** | **0.0000** |
| **72** | 0.988 | +0.65 | 1.0000 |
| 77 | **0.983** | **-1.30** | **0.0000** |
| 78 | **0.988** | **-1.96** | **0.0000** |
| 79 | **0.997** | **-0.72** | **0.0000** |
| 84 | **0.982** | **-0.65** | **0.0002** |
| 97 | **0.991** | **+0.04** | **0.0012** |
| 100 | **0.985** | **-2.41** | **0.0000** |
| **102** | 1 | 0.00 | 1.0000 |
| 104 | **1** | **-6.96** | **0.0000** |
| **105** | 0.986 | +2.68 | 1.0000 |
| **108** | 0.995 | +0.88 | 1.0000 |
| **110** | 0.994 | +1.05 | 1.0000 |
| **114** | 1 | +0.51 | 1.0000 |
| **126** | 0.998 | +1.97 | 1.0000 |
| **131** | 0.998 | +0.53 | 1.0000 |
| **134** | 1 | 0.00 | 1.0000 |
| **135** | 0.985 | +0.03 | 0.9122 |
| 140 | **1** | **-5.41** | **0.0000** |
| **Site** | **Bayes empirical Bayes (probability)** | **Average change in number of carbons** | **p-value** |
| | **g12** | | |
| 6 | **0.955** | **-1.09** | **0.0000** |
| 13 | **0.988** | **-1.87** | **0.0000** |
| **21** | 0.999 | +2.54 | 1.0000 |
| 22 | **1** | **-2.79** | **0.0000** |
| 28 | **0.999** | **-2.71** | **0.0000** |
| 38 | **0.98** | **-0.77** | **0.0000** |
| 44 | **0.992** | **-0.49** | **0.0000** |
| 55 | **1** | **-1.02** | **0.0000** |
| 59 | **0.997** | **-1.61** | **0.0000** |
| **66** | 1 | +1.00 | 1.0000 |
| 72 | **0.999** | **-2.97** | **0.0000** |
| **86** | 0.967 | +0.81 | 1.0000 |
| **88** | 0.999 | +0.02 | 0.5324 |
| **89** | 0.965 | +0.55 | 0.9976 |
| **99** | 0.981 | +0.01 | 0.8037 |
| **110** | 0.97 | -0.39 | 0.0107 |
| 122 | **0.982** | **-3.80** | **0.0000** |
| **125** | 0.97 | +0.67 | 1.0000 |
| **149** | 1 | +1.76 | 1.0000 |
| 152 | **0.983** | **-0.32** | **0.0014** |
| **155** | 0.998 | +0.06 | 0.7210 |

| | | | |
|---|---|---|---|
| **184** | 0.999 | +0.82 | 1.0000 |
| 202 | **0.991** | **-0.37** | **0.0000** |
| **Site** | **Bayes empirical Bayes (probability)** | **Average change in number of carbons** | **p-value** |
| | **g13** | | |
| **18** | 0.97 | +2.22 | 1.0000 |
| **27** | 0.996 | +0.04 | 0.9421 |
| **33** | 0.991 | +0.69 | 0.9986 |
| 44 | **0.985** | **-0.94** | **0.0000** |
| 69 | **0.967** | **-1.95** | **0.0000** |
| **70** | 0.969 | +1.30 | 1.0000 |
| **71** | 0.984 | +1.96 | 1.0000 |
| 72 | **0.998** | **-0.86** | **0.0002** |
| **76** | 0.974 | +0.82 | 1.0000 |
| 78 | **0.967** | **-0.59** | **0.0028** |
| **86** | 0.991 | +0.60 | 0.9994 |
| **94** | 0.991 | +0.41 | 0.4857 |
| **114** | 0.98 | +1.09 | 1.0000 |
| 131 | **0.984** | **-0.22** | **0.0000** |
| 135 | **0.991** | **-1.80** | **0.0000** |
| 143 | **0.964** | **-0.98** | **0.0000** |
| 170 | **0.959** | **-1.00** | **0.0000** |
| 173 | **0.965** | **-1.45** | **0.0000** |
| 174 | **0.977** | **-0.35** | **0.0000** |
| 175 | **0.968** | **-0.66** | **0.0001** |
| **177** | 0.995 | +2.47 | 1.0000 |
| **186** | 0.97 | +1.14 | 0.9999 |
| 231 | **0.99** | **-5.97** | **0.0000** |
| **238** | 0.979 | +1.68 | 0.9999 |
| **242** | 0.988 | +1.42 | 1.0000 |
| **244** | 0.972 | +1.42 | 1.0000 |
| 284 | **0.966** | **-6.68** | **0.0000** |
| 301 | **0.964** | **-2.67** | **0.0000** |
| **302** | 0.977 | +0.47 | 0.9735 |
| 327 | **0.999** | **-3.96** | **0.0000** |
| 337 | **0.967** | **-1.78** | **0.0000** |
| **Site** | **Bayes empirical Bayes (probability)** | **Average change in number of carbons** | **p-value** |
| | **g15** | | |
| **37** | 0.957 | +2.34 | 1.0000 |
| **54** | 0.998 | +4.06 | 1.0000 |
| 76 | **0.954** | **-1.25** | **0.0000** |
| **107** | 0.987 | +0.04 | 0.9073 |

| | | | |
|---|---|---|---|
| **125** | 0.981 | +2.26 | 1.0000 |
| 126 | **0.985** | **-3.30** | **0.0000** |
| **170** | 0.961 | -0.10 | 0.1523 |
| 187 | **0.966** | **-2.96** | **0.0000** |
| **205** | 0.956 | +1.56 | 1.0000 |
| 219 | **0.974** | **-1.75** | **0.0000** |
| 222 | **0.95** | **-1.23** | **0.0000** |
| **302** | 0.984 | +0.87 | 1.0000 |
| **376** | 0.983 | +0.24 | 0.8946 |
| 403 | **0.962** | **-1.96** | **0.0000** |
| 408 | **0.955** | **-1.06** | **0.0000** |
| **413** | 0.961 | +0.91 | 0.9775 |
| 422 | **0.965** | **-0.78** | **0.0000** |
| 427 | **0.957** | **-0.63** | **0.0020** |
| 435 | **0.987** | **-2.52** | **0.0000** |
| **509** | 0.953 | +2.58 | 1.0000 |
| 512 | **0.975** | **-6.30** | **0.0000** |
| **547** | 0.954 | -1.77 | 0.0120 |
| **551** | 0.957 | +1.18 | 1.0000 |
| 574 | **0.968** | **-4.20** | **0.0000** |
| 622 | **0.957** | **-0.52** | **0.0000** |
| 624 | **0.952** | **-2.42** | **0.0000** |
| **684** | 0.987 | +1.93 | 1.0000 |

$Sites in bold font significantly reduce carbon utilization in *Sphingomonadales* with corrected p-values < 0.01
